# Comprehensive metabolome and transcriptome analyses demonstrate divergent anthocyanin and carotenoid accumulation in fruits of wild and cultivated loquats

**DOI:** 10.3389/fpls.2023.1285456

**Published:** 2023-10-13

**Authors:** Wenbing Su, Changqing Zhu, Zhongqi Fan, Mingkun Huang, Han Lin, Xiuping Chen, Chaojun Deng, Yongping Chen, Yidan Kou, Zhihong Tong, Yaling Zhang, Changjie Xu, Shaoquan Zheng, Jimou Jiang

**Affiliations:** ^1^ Fruit Research Institute, Fujian Academy of Agricultural Science, Fuzhou, China; ^2^ Zhejiang Provincial Key Laboratory of Horticultural Plant Integrative Biology/State Agriculture Ministry Laboratory of Horticultural Plant Crop Growth and Development, Zhejiang University, Hangzhou, China; ^3^ Institute of Postharvest Technology of Agricultural Products, College of Food Science, Fujian Agriculture and Forestry University, Fuzhou, Fujian, China; ^4^ Lushan Botanical Garden, Chinese Academy of Sciences, Jiujiang, Jiangxi, China

**Keywords:** pigmentation, carotenoid, anthocyanin, *PSY*, *ANS*, evolution, loquat

## Abstract

*Eriobotrya* is an evergreen fruit tree native to South-West China and adjacent countries. There are more than 26 loquat species known in this genus, while *E. japonica* is the only species yet domesticated to produce fresh fruits from late spring to early summer. Fruits of cultivated loquat are usually orange colored, in contrast to the red color of fruits of wild *E. henryi* (EH). However, the mechanisms of fruit pigment formation during loquat evolution are yet to be elucidated. To understand these, targeted carotenoid and anthocyanin metabolomics as well as transcriptomics analyses were carried out in this study. The results showed that β-carotene, violaxanthin palmitate and rubixanthin laurate, totally accounted for over 60% of the colored carotenoids, were the major carotenoids in peel of the orange colored ‘Jiefangzhong’ (JFZ) fruits. Total carotenoids content in JFZ is about 10 times to that of EH, and the expression levels of *PSY*, *ZDS* and *ZEP* in JFZ were 10.69 to 23.26 folds to that in EH at ripen stage. Cyanidin-3-*O*-galactoside and pelargonidin-3-*O*-galactoside were the predominant anthocyanins enriched in EH peel. On the contrary, both of them were almost undetectable in JFZ, and the transcript levels of *F3H*, *F3’H*, *ANS*, *CHS* and *CHI* in EH were 4.39 to 73.12 folds higher than that in JFZ during fruit pigmentation. In summary, abundant carotenoid deposition in JFZ peel is well correlated with the strong expression of *PSY*, *ZDS* and *ZEP*, while the accumulation of anthocyanin metabolites in EH peel is tightly associated with the notably upregulated expressions of *F3H*, *F3’H*, *ANS*, *CHS* and *CHI*. This study was the first to demonstrate the metabolic background of how fruit pigmentations evolved from wild to cultivated loquat species, and provided gene targets for further breeding of more colorful loquat fruits via manipulation of carotenoids and anthocyanin biosynthesis.

## Introduction

Accumulation of pigments including anthocyanins, chlorophylls, and carotenoids is an essential process regulating plant growth and development, as well as for adjusting to environmental stresses. For example, anthocyanin is a popular pigment enriched in organs like fruit, leaf, and petal to attract pollinating insects and foragers to guarantee successful pollination and seed spread, or oppose herbivores (Tanaka et al., 2008). In addition, high enrichment of these pigments also improves fruit appearance and flavor, and supports human beings with health nutrients. Traditional breeding (Bang et al., 2007), biofortification (Römer et al., 2000), and agronomic protocols (Luan et al., 2020) have been long used to enhance pigment accumulation in plant products. The identification of specific chemical basis and gene regulation of specific pigments accumulated in plant tissues is vital for health pigments improvement.

Carotenoids are mostly C40 terpenoids essential for plant life and human health. These compounds often participate in various biological processes, such as photosynthesis, photomorphogenesis, photoprotection, and development (Nisar et al., 2015). Furthermore, they also serve as precursors for plant hormones (abscisic acid and strigolactone) and for a diverse set of apocarotenoids (Nisar et al., 2015). For human beings, carotenoids are critical components abundantly enriched in diverse foods to supply antioxidants and provitamin A (Römer et al., 2000; Hermanns et al., 2020). These compounds are derived from the isoprene precursors, isopentenyl diphosphate (IPP) and its allylic isomer dimethylallyl diphosphate (DMAPP). To date, dozens of enzymes have been identified with a function in carotenoid biosynthesis and metabolism (Nisar et al., 2015; Hermanns et al., 2020). For fruit production crops, natural variations in *IPI* (I*sopentenyl diphosphate isomerase*), *PSY* (*phytoene synthase*), *CRTISO* (*carotenoid isomerase*), *LCYB* (*lycopene β-cyclase*), *ZEP* (*zeaxanthin epoxidase*), *OR* (*Orange*), and *NCED*/*CCD* (9-*cis*-epoxycarotenoid dioxygenase/*carotenoid cleavage dioxygenase*) genes resulted in diverse colored fruits ranging from white, yellow, pink, orange to red in tomato (Pankratov et al., 2016; Yoo et al., 2023), pepper (Lee et al., 2021), watermelon (Bang et al., 2007; Liu et al., 2021), melon (Tzuri et al., 2015), citrus (Zheng et al., 2019), peach (Falchi et al., 2013), papaya (Wu et al., 2017) and loquat (Fu et al., 2014). These genes encode enzymes that function in the carotenoid biosynthesis and metabolism pathway. Definitely, three IPP units and one DMAPP were condensed into GGPP (geranylgeranyl pyrophosphate) via GGPP synthase (GGPPS). Condensation of two GGPP molecules by PSY led to the formation of phytoene (the first carotenoid compound), PDS (phytoene desaturase) and Z-ISO (ζ-carotene isomerase), then phytoene was introduced into ζ-carotene. ZDS (ζ-carotene desaturase) and *CRTISO* catalyzed phytoene into red-pigmented lycopene. Lycopene was further cyclized by *LCYB* and converted to orange-pigmented γ- and β-carotene. BCH (β-carotene hydroxylase) then catalyzed β-carotene to form yellow-pigmented β-cryptoxanthin and zeaxanthin, and zeaxanthin was further converted into antheraxanthin and violaxanthin by ZEP. NSY (neoxanthin synthase) subsequently catalyzed violaxanthin into neoxanthin. Finally, neoxanthin and some other carotenoids were turned into apocarotenoids by NCED/CCD (Nisar et al., 2015; Hermanns et al., 2020; Sun et al., 2022). Function or expression variations of these genes might regulate carotenogenic metabolic flux to abundantly enrich specific carotenoid metabolites in above-mentioned fruit crops, which subsequently resulted into different colored fruits.

Anthocyanins, a clade of flavonoids, are ubiquitous plant secondary metabolites participating in attracting insect pollinators and seed dispersers (Tanaka et al., 2008), stress protection (Zhao et al., 2019; Sun et al., 2023) as well as supporting strong medicinal value for humans (Giampieri et al., 2018). Generally, anthocyanins are synthesized by the phenylpropanoid pathway and downstream flavonoid pathway. Primarily, PAL (phenylalanine ammonia lyase), C4H (cinnamate 4-hydroxylase) and 4CL (4-coumarate:CoA ligase) enzymes in the phenylpropanoid pathway convert phenylalanine into 4-coumaroyl-CoA (Tanaka et al., 2008). Then, CHS (chalcone synthase), CHI (chalcone isomerase), F3H (flavanone 3-hydroxylase), F3’H (flavonoid 3′-hydroxylase), F3’5’H (flavonoid 3′5′-hydroxylase), DFR (dihydroflavonol 4-reductase), ANS (anthocyanidin synthase), UGT (uridine diphosphate-dependent glucosyltransferase), and other modification enzymes from the flavonoid pathway catalyze 4-coumaroyl-CoA into anthocyanin and other flavonoid compounds (Tanaka et al., 2008). Constitute and content of the flavonoid compound enriched in plant tissue depend on the capacities of these enzymes. CHS is the first committed step of flavonoid biosynthesis and plays vital roles in flavonoid biosynthesis of apple skin and flesh (Dare et al., 2013). CHI catalyzes the conversion of chalcones to flavanones, as an example, overexpression of *DcCHI1* (isolated from Dragon’s blood) in tobacco significantly increased flavonoid production (Zhu et al., 2021). F3H is a key enzyme in directing carbon flow towards the biosynthesis of 3-hydroxylated flavonoids, since the loss of *FvF3H* function blocks anthocyanin biosynthesis in strawberry fruits (Xu et al., 2023). F3’H catalyzes the introduction of an additional hydroxyl group in the B-ring of various flavonoids, and the loss of function of the F3’H in *Arabidopsis* inhibits dihydroquercetin production, and leads to overaccumulation of kaempferol-3-rhamnoside (Niñoles et al., 2023). ANS is a late key enzyme in the flavonoid pathway which catalyze the colorless leucoanthocyanidins to red-, purple- and orange red-colored anthocyanidins. Loss of function of ANS proteins in raspberry and eggplant both resulted in loss of visible and detectable anthocyanin pigments (Rafique et al., 2016; Chen et al., 2018). UGTs (UDP-glycosyltransferases), include UDP-glucose (UDP-Glc), UDP-galactose (UDP-Gal), and UDP-rhamnose (UDP-Rha), are the largest group of plant glycosyltransferases catalyzing glycosylation of flavonols, which occurs during the later stages of flavonol biosynthesis (Ren et al., 2022). Among these, MdUGT78T2 functions in transferring galactosyl from UDP-Gal to flavonols to produce major flavonoid glycol conjugates (quercetin 3-*O*-galactoside and cyanidin-3-*O*-galactoside) in apple fruit (Clayton-Cuch et al., 2023).

**Figure 1:**
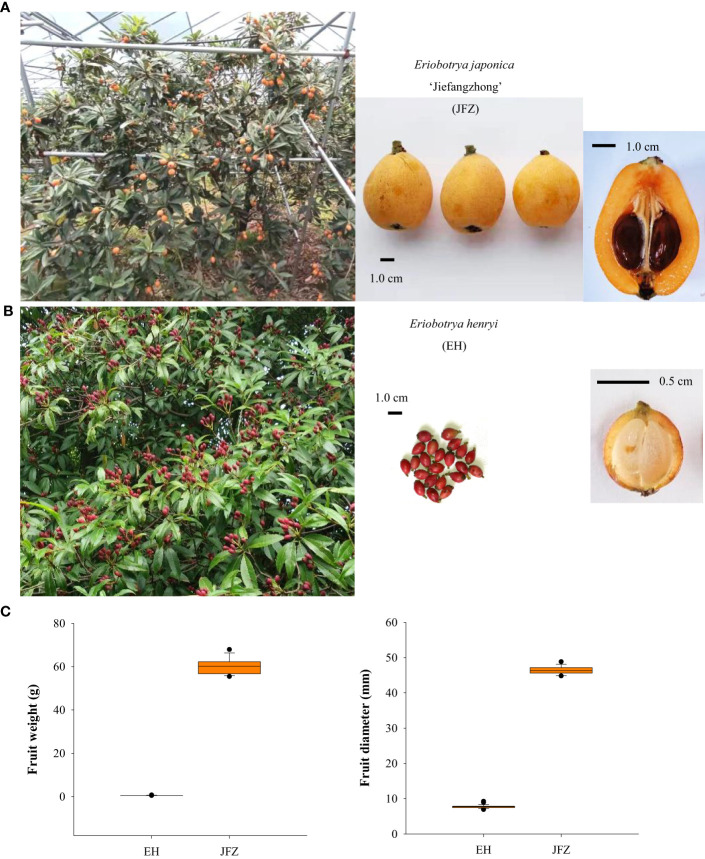
Trees and fruits of cultivated and wild loquats. **(A)** Large tree and orange-colored fruits of the ‘Jiefangzhong’ cultivar. **(B)** Small tree and red-colored fruits of *Eriobotrya henryi.* **(C)** Fruit weight and fruit diameter of JFZ and EH.

Loquat (*Eriobotrya japonica*) is a distinctive subtropical fruit tree (apple subfamily, Rosaceae) native to South-West China, which supports human with delicious and nutritious fruits from late spring to early summer (Su et al., 2021b). Carotenoids are one of the most important nutrients in loquat fruits, and cultivated loquats are commonly classified into white-, yellow-, orange-, and orange-red fleshed groups, due to differences in carotenoid amount (Zhou et al., 2007). Previously, carotenoid quality and quantity assays identified β-carotene as the predominant pigment of cultivated loquat and segment mutations in *EjPSY2A* coding region principally confers to the variations of total carotenoid content and flesh color (Fu et al., 2012; Fu et al., 2014). While cultivated loquat usually harbors yellow or orange fruit, fruits from the wild *E. henryi* are generally in red or purple color (**Figure 1**) (Su et al., 2021b). However, whether the red and purple colors are derived from higher accumulation of the well-known carotenoids like the cultivated loquat or from the accumulation of other pigments, and how the colorful compounds were enriched in fruit tissues, are still unknown. Here, we performed integrative analyses of targeted metabolomics and transcriptomics to identify key pigment compounds underlying loquat fruit color variation and evolution, and screened candidate important structure genes responsible for *in vivo* biosynthesis of these pigments in loquat fruits. All these data collectively revealed the metabolic basis of fruit color evolution and shed light on the breeding for more colorful and nutritious loquat fruits.

## Materials and methods

### Plant materials and growth conditions

Ripen fruits of cultivated loquat were collected from ‘Jiefangzhong’ (JFZ) trees planted in the National Germplasm Bank of Loquat (Fuzhou, Fujian, China) on 15th April, 2021. Trees of the wild loquat species, *Eriobotrya henryi* (EH), were native grown in Kunming Botanical Garden (Kunming, Yunnan, China), and red-colored ripen fruits were collected on 24th June, 2021. Fruit cortex samples, including pulp and peel tissues, were collected and immediately frozen in liquid nitrogen, thereafter stored at -80°C, three biological repeats were performed for pigment analysis and RNA library construction, five fruits were used in each repeat. Fruits from green, color breaker and ripen stages were collected from JFZ and EH trees during the fruiting season of 2023 for gene expression analyses.

### Carotenoids extraction and targeted metabolome analysis

Frozen loquat fruit tissues were freeze-dried for 24 h with a freeze dryer (Labconco, America) and ground at 30 Hz into fine powder using a mixer mill. The fruit carotenoids were extracted as previously described (Song et al., 2023) with some modifications. 50 mg fruit powder was added into 500 μL mixed solution of n-hexane: acetone: ethanol (1:1:1, v/v/v), and 10 μL carotenoids internal standard (IS) mixed solution (20 μg/mL) was added into the extracting tube for the quantification. Then they were mixed for 20 min at room temperature with the vortex mixer, centrifuged at 4°C at 12000 r/min for 5 min and the supernatants were collected. The above steps were repeated twice until the samples is colorless. Then the supernatant was evaporated to dryness, and reconstituted in mixed solution of Methanol: Methyl tert-butyl ether (1:1, v/v). The obtained liquid was filtered through 0.22 μm membrane filter (Biosharp) before LC-MS/MS analysis.

The carotenoid extracts were analyzed using UPLC-MS/MS (ExionLC™ AD, UPLC, https://sciex.com.cn/; Applied Biosystems 6500 Triple Quadrupole, MS, https://sciex.com.cn/) system. The analysis was performed according to Zhou et al. (2020). The YMC C30 column (3 µm, 100 mm×2.0 mm) was used, with temperature at 28°C and flow rate at 0.8 ml/min. 2 μL extract was injected for each detection. The mobile phase was made up of phase A (methanol: acetonitrile (1:3, v/v) with 0.01% BHT and 0.1% formic acid) and phase B (methyl tert-butyl ether with 0.01% BHT). The elution gradient began with 0% phase B from 0 min to 3 min, then increased to 70% at 3-5 min, then increased to 95% at 5-9 min, and finally ramped back to 0% at 10-11 min.

A QTRAP^®^ 6500+ LC-MS/MS System, equipped with an APCI Heated Nebulizer, was operated in positive ion mode and controlled by Analyst 1.6.3 software (Sciex). The following source operation parameters were used: ion source, APCI+; source temperature, 350°C; curtain gas (CUR), 25.0 psi; and collision gas (CAD). Carotenoids data acquisitions were performed using Analyst 1.6.3 software (Sciex). The integrated peak area of each carotenoid detected in the samples was substituted into the linear equations of standard curves for content calculation (see **Table S1** for the standard curves). Three biological repeats were used for each sample.

### Anthocyanins extraction and quantification

Total anthocyanin was extracted as formerly performed (Yi et al., 2021). 50 mg vacuum freeze-dried fine fruit tissue powder for each sample was weighted and added into 0.5 mL methanol/water/hydrochloric acid (500:500:1, V/V/V). The samples were vortexed for 30 s then immersed in methanol for 30 min. After six rounds of vortexing-immersing, these samples were placed in a 4°C refrigerator for overnight extraction. Samples were finally centrifuged at 12,000 g for 10 min under 4°C. The supernatants were collected, and filtrated with a microporous membrane (0.22 μm pore size) into injection bottles before subsequent UPLC-MS/MS analysis.

Anthocyanin UPLC-MS/MS analysis was performed according to Huang et al. (2019). The anthocyanins in two kinds of loquat fruits were analyzed by UPLC-MS/MS system comprising the SCIEX ultra-performance liquid chromatography, the Applied Biosystems 6500 Triple Quadrupole mass spectrometry, and the Waters ACQUITY BEH C18 column (1.7 µm, 2.1 mm×100 mm). The mobile phase was made up of phase A (0.1% formic acid in ultrapure water) and phase B (0.1% formic acid in acetonitrile). The column temperature was 40°C, with a 0.35 ml/min flow rate. The sample injection volume was 2 μL. The elution gradient began with 5% phase B at 0 min, then increased to 50% at 6 min, and the proportion of phase B increased to 95% at 12 min and was maintained for 2 min. Anthocyanins contents were measured using Analyst 1.6.3 software based on the AB Sciex QTRAP 6500 LC-MS/MS platform.

The ESI source operation parameters were following: ion source, ESI+; source temperature 550°C; ion spray voltage (IS) −4500 V (negative ions) and 5500 V (positive ions); curtain gas (CUR) was set at 35 psi. Anthocyanins were analyzed using multiple reaction monitoring (MRM). Multiquant 3.0.3 software (Sciex) was used to quantify all metabolites. The m/z range used in the LC-MS/MS analysis was 50–1250 Da. The integrated peak area of each detection was substituted into the linear equations of anthocyanins standard curves for sample level calculation (see **Table S2** for the standard curves). Three spears were used for each repeat.

### RNA extraction and cDNA preparation

Total RNA of these samples was extracted from fine powder fruit tissues as formerly descripted (Su et al., 2017) with EASYspin Plus plant RNA kit (Aidlab, China). The 1.5% agarose gel electrophoresis was performed to evaluate the integrity of the RNA, and the RNA concentration and purity were then assayed with a NanoDrop ND-1000 spectrophotometer (NanoDrop Technologies, Montchanin, DE, USA). A PrimeScript™ RT reagent Kit with genome DNA wiper (TaKaRa, Japan) was then used to synthesize the first-strand cDNA of the plant samples according to the manufacturer’s instructions.

Library construction was performed by BioMarker Co., LTD (Beijing, China) using NEBNext^®^ UltraTM RNA Library Prep Kit and sequenced with the Illumina HiSeq 2500 platforms, and 150 bp paired-end reads were then generated. Low quality reads were removed from the data sets using Fastp v0.19.3. The high quality clean reads were aligned to the JFZ reference genome (Su et al., 2021a) released at https://db.cngb.org/search/project/CNP0001531/with HISAT v2.1.0 software. The transcripts were assembled as formerly performed (Trapnell et al., 2010). Then the transcriptome data were analyzed as previously performed by (Peng et al., 2022). Correlation assessment of sample replicates was performed with edgeR (Robinson et al., 2010). DESeq2 was used to estimate the differential expressed genes (DEGs) among fruit samples with more than 2-fold change as well as FDR (false discovery rate) < 0.01. Gene function was annotated by aligning the proteins of each gene to the following databases: GO (http://www.geneontology.org/), KEGG (http://www.genome.jp/kegg/), KOG (http://www.ncbi.nlm.nih.gov/KOG/), NR (ftp://ftp.ncbi.nih.gov/blast/db/), Pfam (http://pfam.xfam.org/), Swiss-Prot (http://www.uniprot.org/). The amino acid sequences from Arabidopsis (listed in **Table S7**) were used to BLAST against the loquat genome to identify carotenoid and anthocyanin biosynthesis structure gene homologues as formerly performed with e-value<1e-5, Number of Hits>50 and Number of Alignment>50 (Su et al., 2017).

### Quantitative real-time PCR assays

Total RNA of fruits from three developmental stages were prepared as above performed for RNA-seq library construction. Quantitative real-time RT-PCR analysis was performed as previously done (Su et al., 2019). Integrated DNA Technologies software (https://sg.idtdna.com/pages) was applied to design the primers for quantitative real-time polymerase chain reaction (qRT-PCR). *EjACT2* (AB710173.1) was used as the reference gene. Primer information for all the interested biosynthesis genes were listed in **Table S8**. Each value confers to the mean of three biological replicates captured by the LightCycler480 Q-PCR system (Roche, Sweden) using iTaq™ universal SYBR Green Supermix purchased from Bio-Rad.

### DNA extraction and *PSY2A* genotyping

Genomic DNA samples were extracted from young leaves of JFZ and EH with a M5 CTAB plant gDNA extract Kit (Mei5 Biotechnology, Beijing, China) according to the user’s protocol. Genotyping of *PSY2A*, a candidate gene formerly confirm by (Fu et al., 2014) to underlie flesh color and carotenoid variation of cultivated loquat fruits, with forward primer: 5’-ATTTGCCAACTACCACTGCTTTCA-3’ and reverse primer: 5’-TACACCACATAAGAAACAAGCA-3’. The PCR amplicons were monitored on 1.5% agarose gels.

## Results

### Fruit appearance of wild and cultivated loquats

In general, fruits of the cultivated *Eriobotrya japonica* are mostly in yellow to orange-red colors, globose to obovate shape, and 2.0-5.0 cm fruit size. For example, one of the traditional main cultivars, JFZ, is about 5 cm in transverse diameter and both its peel and flesh are orange (**Figures 1A, C**). While the *E. japonica* is domesticated for fruit production, most wild loquat species set small fruits with very thin flesh and cannot meet consumption demands. Among these species, *E. henryi* possess attractive red pigmented fruits in oval shape, and the flesh from their fruit is light yellow or white (**Figures 1B, C**).

### Carotenoid components accumulated in peel of wild and cultivated loquat fruits

**Figure 2:**
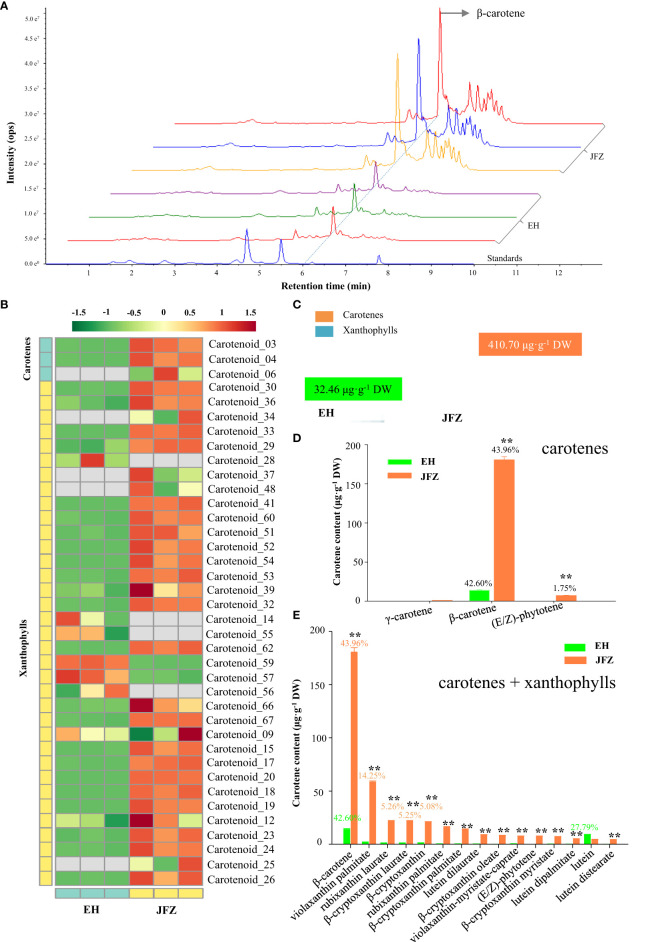
Carotenoid components of cultivated and wild loquat fruit peels. **(A)** Chromatograms of β-carotene and other carotenoids enriched in JFZ and EH by UPLC-MS/MS. **(B)** Heat map of carotenoid metabolite clusters. **(C)** Total carotenoid metabolite content. **(D)** Detailed carotene compound levels of the two species. **(E)** Top-15 carotenoid compounds in the two species. ** indicates P < 0.01 by Student’s t-test.

To understand what pigments contribute to the color changes of the two loquat species, targeted carotenoid metabolomics was firstly performed on peel of ripen fruits. Obviously, more and higher carotenoid compound peaks were detected in JFZ, among which β-carotene is the highest at 6.21 min by UPLC-MS/MS (**Figure 2A**). In total, 38 carotenoid components were identified from the fruit peel of the two loquat species, including β-carotene, violaxanthin palmitate, rubixanthin laurate, β-cryptoxanthin laurate, β-cryptoxanthin, rubixanthin palmitate, β-cryptoxanthin palmitate, lutein dilaurate, β-cryptoxanthin oleate, violaxanthin-myristate-caprate, (E/Z)-phytoene, β-cryptoxanthin myristate, lutein dipalmitate, lutein, etc. (**Figure 2B**, **Tables S1**, **2**). And the majority of these compounds existed in both of the two species (little in EH), while the total carotenoid content in JFZ (410.70μg·g^-1^) was about twelve folds higher to that in EH (**Figure 2C**). For both of them, β-carotene accounted for more than 42% of the total carotenoids, and β-carotene content in JFZ is thirteen folds to that in EH (**Figure 2D**). Metabolites derived from γ-carotene (including β-carotene, β-cryptoxanthin, violaxanthin, rubixanthin and zeaxanthin) accounted for more than 90% of the total carotenoids in JFZ. The top 15 highly enriched carotenoids compounds, except for lutein, were significantly accumulated at higher levels in JFZ (**Figure 2E**).

### Identification of anthocyanins components enriched in peel of wild loquat fruits

**Figure 3:**
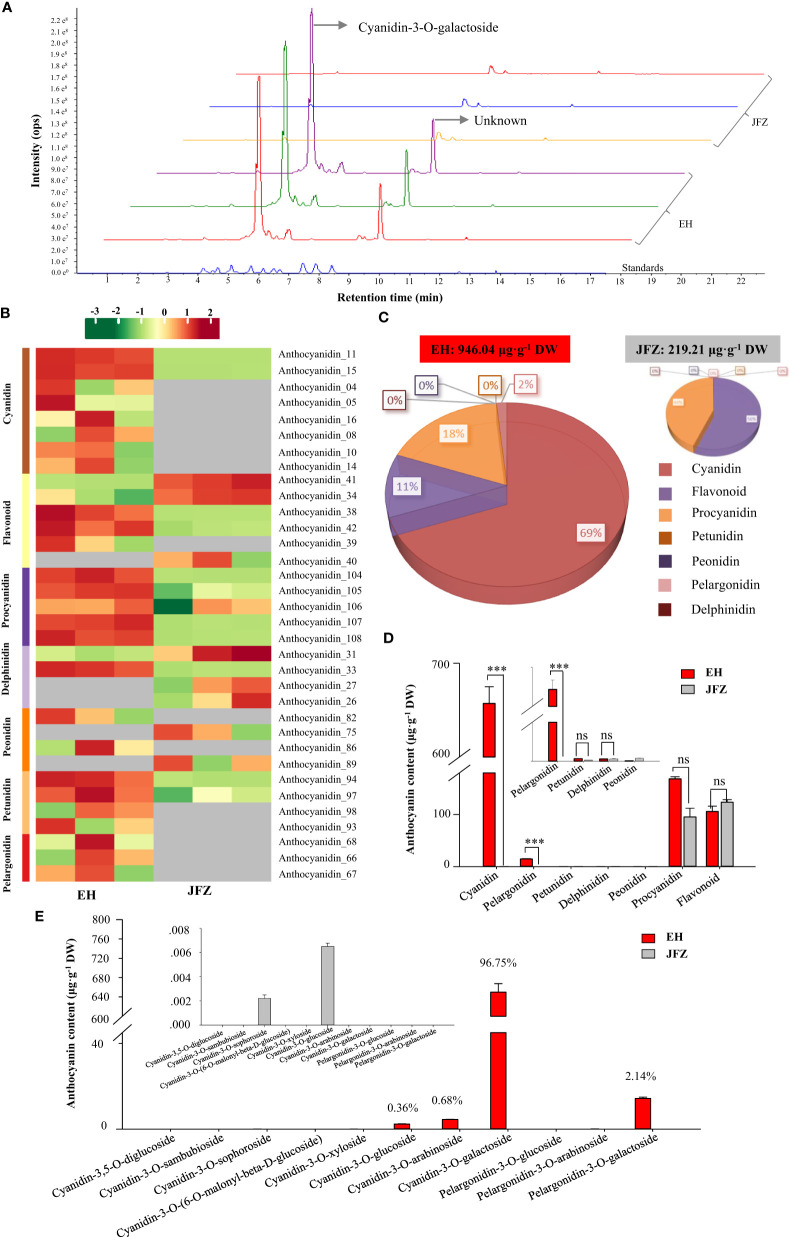
Anthocyanin components in peel of cultivated and wild loquat fruits. **(A)** Chromatograms of cyanidin-3-*O*-galactoside and other anthocyanins in JFZ and EH by UPLC-MS/MS. **(B)** Heat map of flavonoids metabolite clusters. **(C)** Total flavonoids metabolite content in JFZ and EH. **(D)** Cyanidin, pelargonidin, petunidin, delphinidin, peonidin, procyanidin and flavonoid contain of the two species. **(E)** Detail anthocyanin compound levels of the two species. *** indicates P < 0.001 and NS indicates no significant difference by Student’s t-test.

Lower carotenoid accumulation in above detection confirmed that carotenoid was not the major pigment explaining the deeper color of EH fruits. Since anthocyanins are the major pigments for the red color of apple and pear fruits (both of them are relatives of loquat from the apple subfamily) (Espley et al., 2007; Ni et al., 2023), we then carried out flavonoids metabolomics to understand the metabolite basics for red coloring of wild loquat fruits. The UPLC-MS/MS detection showed abundant and high anthocyanin compound peaks in peel of EH fruits, among which cyanidin-3-*O*-galactoside was the highest at 5.11 min, however the second highest peak at 9.48 min is an unknown compound very similar to naringenin-7-*O*-glucoside (9.16 min) (**Figure 3A**). In total, 34 flavonoids components were identified from the two loquat species, including 8 cyanidins, 6 flavonoids, 5 procyanidins, 4 delphinidins, 4 peonidins, 4 petunidins, and 3 pelargonidins (**Figure 3B**, **Tables S3**, **4**). In sum, the total flavonoids in EH fruits were 4.31 folds to that in JFZ fruits while it is unexpected that the cultivated JFZ contained similar levels of flavonoid in mature fruit compared to the wild loquat (**Figures 3C, D**). Most of the anthocyanin compounds (cyanidin, delphinidin, peonidin and pelargonidin) were trace or undetectable in peel of JFZ, and cyanidin compounds accounted for 69% of total flavonoids in EH (**Figure 3C**). Cyanidin (thousands of times higher) and pelargonidin levels were significantly higher accumulated in EH compared with that in JFZ (**Figure 3D**). Cyanidin-3-*O*-galactoside (659.07 μg·g-1), cyanidin-3-*O*-arabinoside (4.47 μg·g-1) and cyanidin-3-*O*-glucoside (2.53 μg·g-1) accounted for more than 97% of the total anthocyanin enriched in EH while pelargonidin-3-*O*-galactoside (14.56 μg·g-1) accounted for 2.14% (**Figure 3E**).

### Transcriptome sequencing data assembly and annotation

Transcriptome sequencing analysis was carried out on mature JFZ and EH fruit peel samples. A total of 29.47 Gb raw reads with sequencing error rate lower than 0.03% were obtained from the sequencing of all six libraries, and the biological replicates of different samples were clustered together to show high reproducibility (**Figure S1A**). A total of 28.30 Gb clean reads were obtained with Q20>97.68%, Q30>93.42% after filtering and the GC content ranged from 46.87% to 47.26% (**Table S5**). More than 91% of the clean reads from JFZ could be unique mapped to reference genome, while only about 77% for that of the wild EH (**Table S6**). Differentially expressed gene (DEG) analysis identified 12,888 DEGs between the two ripen fruit samples, among these 6113 were down-regulated in JFZ and 6775 were up-regulated (**Figure S1B**). KEGG, and GO databases annotation showed that lots of DEGs were significantly enriched in pigment metabolism related pathways like ‘pigment metabolic process’, ‘pigment biosynthetic process’, ‘phenylpropanoid biosynthesis’, ‘isoflavonoid biosynthesis’ and ‘terpenoid biosynthesis’ (**Figures S1C, D**). To further explore the mechanism of carotenoids and flavonoids accumulating in loquat fruits, the expression patterns of enzyme encoding genes in the carotenoid and flavonoid pathways were analyzed.

### Expression analysis of carotenoid metabolic pathway genes

**Figure 4:**
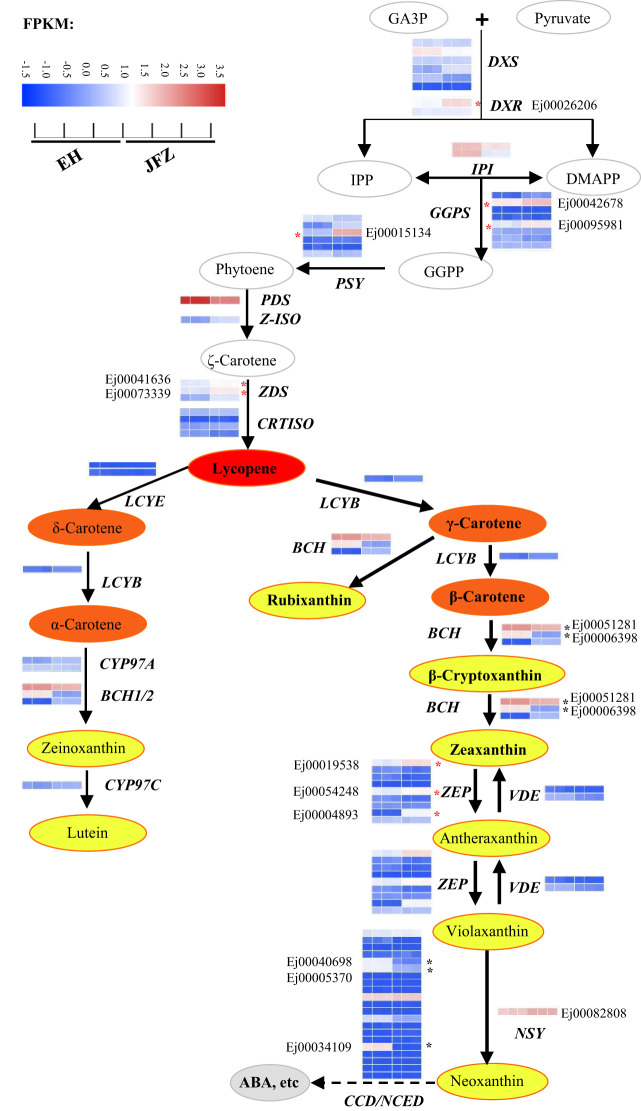
Expressions of carotenoid biosynthesis pathway genes in peel of cultivated and wild loquat fruits. * indicate significant different expression between JFZ and EH. BCH, β-carotene hydroxylase; CYP97, cytochrome P450 carotene hydroxylase; CRTISO, carotenoid isomerase; DMAPP, dimethylallyl diphosphate; DXS, 1-deoxy-D-xylulose 5-phosphate synthase; DXR, 1-deoxy-D-xylulose 5-phosphate reductoisomerase; GA3P, glyceraldehyde 3-phosphate; GGPS, geranylgeranyl diphosphate synthase; IPI, isopentenyl diphosphate isomerase; IPP, isopentenyl diphosphate; LCYB, lycopene β-cyclase; LCYE, lycopene ϵ-cyclase; NCED/CCD, 9-*cis*-epoxycarotenoid dioxygenase/carotenoid cleavage dioxygenase; NSY, neoxanthin synthase; PDS, phytoene desaturase; PSY, phytoene synthase; VDE, violaxanthin de-epoxidase; ZDS, ζ-carotene desaturase; ZEP, zeaxanthin epoxidase; Z-ISO, ζ-carotene isomerase.

The amino acid sequences of carotenoid metabolic enzymes from Arabidopsis were first used to identify carotenoid metabolism pathway genes in loquat. A total of 81 genes were identified, including six *DXS*, two *DXR*, two *HDS*, two *HDR*, two *IPI*, eight *GGPS*, six *PSY*, one *PDS*, one *Z-ISO*, three *ZDS*, four *CRTISO*, one *LYCB*, two *LCYE*, three *BCH*, two *CYP97A*, one *CYP97B*, one *CYP97C*, nine *ZEP*, two *VDE*, twenty-one *NCED*/*CCD* and one *NSY* (**Figure 4**, **Table S7**). Many of these genes were differentially expressed in peel between JFZ and EH. Among them, the expression patterns of *DXR* (Ej00026206), *GGPS* (Ej00042678 and Ej00095981), *PSY* (Ej00015134), *ZDS* (Ej00041636 and Ej00073339), *BCH* (Ej00051281 and Ej00006398), *ZEP* (Ej00019538, Ej00054248 and Ej00004893) and *NCED*/*CCD* (Ej00040698, Ej00005370 and Ej00034109) were associated with carotenoid metabolites changes between JFZ and EH (**Figure 4**). Among these, the expression levels of *DXR*, *GGPS*, *PSY*, *ZDS* and *ZEP* were significantly up-regulated while those of *NCED*/*CCD* were significantly down-regulated in JFZ (**Figure 4**).

### qRT-PCR validated key carotenoid metabolic structural genes

**Figure 5:**
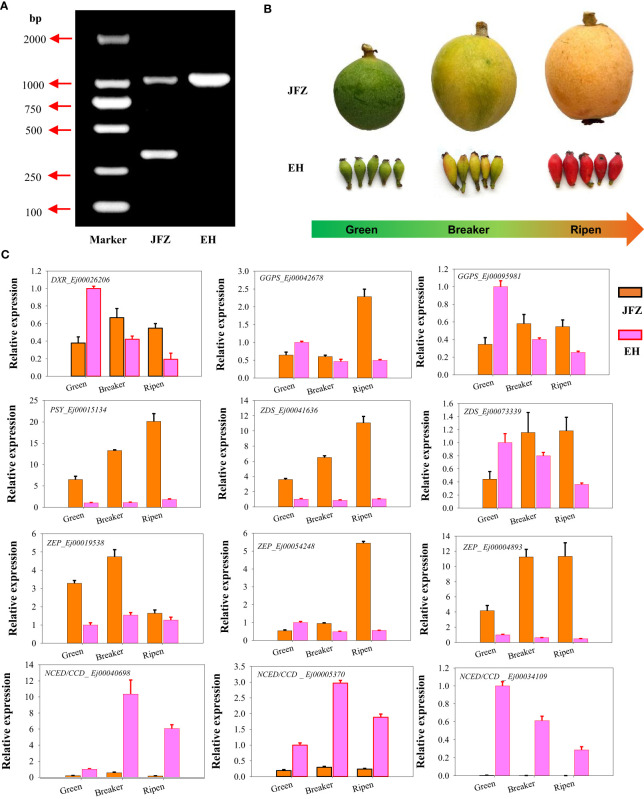
Genotyping and gene expression analyses of carotenoid metabolism genes in peel of cultivated and wild loquat. **(A)** *EjPSY2A* genotyping. **(B)** Fruit samples from green-, color breaker- and ripen developmental stages of JFZ and EH. **(C)** Expression patterns of significantly expressed genes, identified by RNA sequencing, at three key fruit development stages.

DNA genotyping showed that JFZ is an *EjPSY2A-EjPSY2A^d^* heterozygous plant while EH is *EjPSY2A-EjPSY2A* homozygous species (**Figure 5A**). This result indicated that the weak carotenoid contents in EH were not caused by the loss of PSY enzyme function as white-fleshed cultivars performed. Instead, it may be induced by the difference in gene expression levels. To further confirm whether the above-mentioned significantly expressed genes contributed to the carotenoid accumulation divergence of different loquat species, we then collected fruit samples from orange-, red- and purple-coloring varieties at green-, color breaker- and ripen stages for gene expression analyses (**Figure 5B**). Gene expression assays showed that *DXR*, *GGPS*, *PSY*, *ZDS* and *ZEP* were significantly up-regulated from green to color breaker and/or ripen stages, and expressed highest in JFZ (**Figure 5C**). While *DXR*, *GGPS* and *ZDS* were obviously down-regulated in EH as fruits ripening. On the contrary, the expression levels of both of the active *NCED*/*CCD*s (*Ej00040698* and *Ej00005370*) were increased during fruit maturation in both of the two varieties, and they expressed at higher levels in EH than in JFZ. In addition, the transcript levels of *PSY* (*Ej00015134*), *ZDS* (*Ej00041636*) and *ZEP* (*Ej00004893*) in JFZ were more than 5.8 folds to that of EH. Meanwhile, the transcript levels of *NCED*/*CCD*s (*Ej00040698*, *Ej00005370* and *Ej00034109*, function in carotenoids degradation) in EH were 7.88~460.77 folds higher to that in JFZ during fruit coloring.

### Expression analysis of anthocyanin biosynthesis enzyme encoding genes

**Figure 6:**
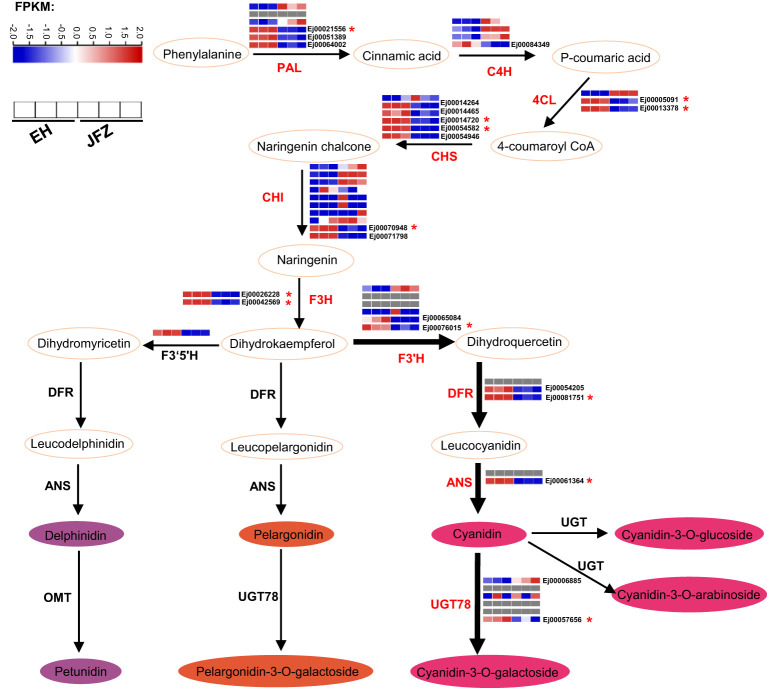
Key enzyme encoding genes identified in the anthocyanin biosynthesis pathway and their expression patterns in peel of JFZ and EH. Gray rectangles indicated null expression detected in the fruits. 4CL, 4-coumarate:CoA ligase; ANR, Anthocyanidin reducase; ANS, Anthocyanidin synthase; C4H, cinnamate 4-hydroxylase; CHI, chalcone isomerase; CHS, chalcone synthase; DFR, dihydroflavonol 4-reductase; F3H, flavanone 3-hydroxylase; F3’H, flavonoid 3′-hydroxylase; F3’5’H, flavonoid 3′5′-hydroxylase; MT, methyltransferase; PAL, phenylalanine ammonia lyase; UGT, uridine diphosphate-dependent glucosyltransferase.

55 enzyme encoding genes (six *PAL*, four *C4H*, three *4CL*, six *CHS*, ten *CHI*, three *F3H*, six *F3’H*, three *DFR*, two *ANS*, six *UGT78*, three *FNS*, one *F3’5’H* and three *LAR*) throughout the anthocyanin and flavonoid biosynthesis pathway were identified (**Figure 6**, **Table S7**). Transcriptome data showed that the expression patterns of *UGT78* (Ej00006885 and Ej00057656), *ANS* (Ej00061364), *DFR* (Ej00081751 and Ej00054205), *F3’H* (Ej00076015 and Ej00065084), *F3H* (Ej00042569 and Ej00026228), *CHI* (Ej00071798 and Ej00070948), *CHS* (Ej00014264, Ej00014720, Ej00054582, Ej00014465 and Ej00054946), *4CL* (Ej00013378 and Ej00005091), *PAL* (Ej00021556, Ej00051389 and Ej00064002) were notably associated with the higher anthocyanin accumulation in peel of EH (**Figure 6**, **Figure S2**). Among these, the higher transcript levels of *UGT78*, *DFR*, *F3’H*, *F3H*, *CHI*, and *CHS* were positively associated with high cyanidin-3-*O*-galactoside and pelargonidin-3-*O*-galactoside in the red-colored EH (**Figure 6**).

### qRT-PCR validated key anthocyanin biosynthesis enzyme encoding genes

**Figure 7:**
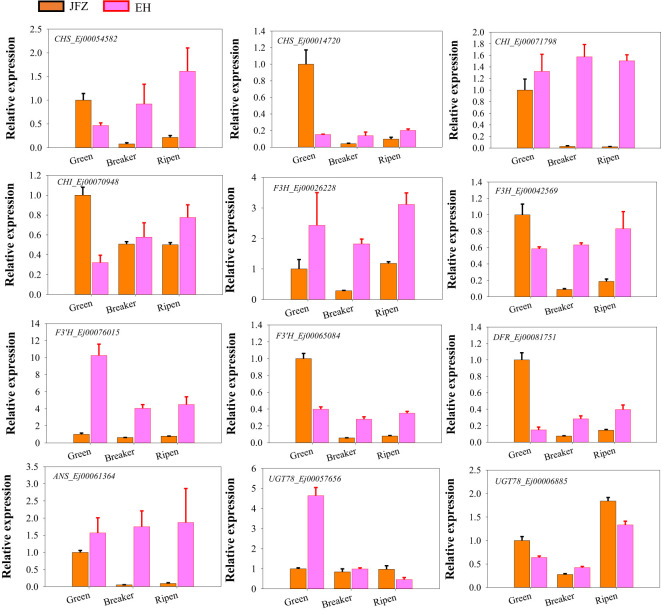
Expression patterns of significantly expressed anthocyanin biosynthesis genes in peel of fruits at three developmental stages.

To further investigate whether the above identified genes contributed to anthocyanin accumulation in loquat fruits, qRT-PCR was carried out to verify their expression patterns of genes in the last seven steps of anthocyanin biosynthesis pathway as formerly performed for carotenoid metabolic genes. The expression data showed that *CHS* (Ej00054582 and Ej00014720), *CHI* (Ej00071798), *F3H* (Ej00026228 and Ej00042569), *F3’H* (Ej00065084), *DFR* (Ej00081751), *ANS* (Ej00061364), and *UGT78* (Ej00006885) were significantly up-regulated in peel of both red-colored EH and purple-colored ES while down-regulated in JFZ as fruits ripen. Another *F3’H* (Ej00076015) was sharply down-regulated from green stage to ripen stage in JFZ, while gently in EH. Moreover, the transcript levels of all these genes were significantly higher in EH than in JFZ (**Figure 7**). The expression levels of *ANS* (Ej00061364), *F3H* (Ej00042569), *F3’H* (Ej00065084), *CHS* (Ej00054582) and *CHI* (Ej00071798) in EH were notably 7.4~8.9, 4.4~7.0, 4.4~4.9, **7.5~27.0** and 55.0~73.1 folds to that in JFZ as fruits start coloring. In addition, the expression level of *UGT78* (Ej00057656) in EH was more than 4.6 folds to that in JFZ.

## Discussion

### Evolution of anthocyanin and carotenoid biosynthesis underlies fruit color variation from ancient loquat to modern cultivar

Commonly, cultivated loquat fruits are classified into white-, yellow-, orange-, and orange-red fleshed groups, due to variations in carotenoid accumulation capabilities of their fruits. Sadana (1949) first revealed that β-carotene is the predominant pigment positively associated with fruit color of cultivated loquats. Then, 23 (Zhou et al., 2007), 25 (De Faria et al., 2009) and 30 (Hadjipieri et al., 2017) carotenoid compounds were identified via HPLC and HPLC-PDA-MS/MS. With violaxanthin palmitate, rubixanthin laurate, β-cryptoxanthin laurate, β-cryptoxanthin, rubixanthin palmitate, β-cryptoxanthin palmitate, lutein dilaurate, β-cryptoxanthin oleate, violaxanthin-myristate-caprate, β-cryptoxanthin myristate and lutein dipalmitate newly identified here, we identified the most carotenoid constituents (38 molecules) from loquat fruit via UPLC-MS/MS (**Figures 2A, B**, **Table S1**). In accordance with former researches in loquat (Zhou et al., 2007; De Faria et al., 2009; Fu et al., 2012), β-carotene was found to be the most abundant compound in both JFZ and EH fruits (**Figure 2**). β-Carotene is also the most abundant carotenoids of apple (Ampomah-Dwamena et al., 2012) and apricot (Zhou et al., 2020), which greatly contributed to their flesh pigmentation. In contrast, lutein and β-cryptoxanthin were the main carotenoid components of peach (Wu et al., 2022) and cherry (Demir, 2013). β-carotene, violaxanthin palmitate and rubixanthin are the top three carotenoids detected in the cultivated JFZ, while lutein is the second most carotenoid of EH (**Figure 2E**). These results suggest that Rosaceae fruit trees are conserved in carotenoid production. Different accumulation capacity of each component, and fold changes of the major carotene constitutes are responsible for flesh color variation of these fruits. The variations in contents of major carotenoid, β-carotene and β-cryptoxanthin, were discovered to underlie the flesh color difference between white and orange-red fleshed cultivars (Zhou et al., 2007; De Faria et al., 2009; Fu et al., 2012). The 10-fold variation of β-carotene and total carotenoid results in white-fleshed wild loquat fruits and orange-fleshed JFZ fruits (**Figure 2**).

It has also been well known that the accumulation of carotenoid compounds results in yellow, orange and orange-red color of loquat fruit (Zhou et al., 2007; Fu et al., 2012; Fu et al., 2014). However, the chemical compounds responsible for red and purple pigmentation of wild loquat remain unclear. Anthocyanin is another types of pigment confers to red pigmentation on plant organs and greatly benefit plant pollination, seed dispersal and so on. Notably, some anthocyanins were abundantly enriched in fruits including apple (Clayton-Cuch et al., 2023), pear (Ni et al., 2023), peach (Cheng et al., 2014), citrus (Huang et al., 2019), grape (Ono et al., 2010), waxberry (Ren et al., 2022), litchi (Li et al., 2016) and longan (Yi et al., 2021) to improve their commodity and bioactivity value. In the present study, 34 flavonoid compounds including 8 cyanidin, 6 flavonoid, 5 procyanidin, 4 delphinidin, 4 peonidin, 4 petunidin and 3 pelargonidin were identified by UPLC-MS/MS, and anthocyanins were identified in *Eriobotrya* genus for the first time (**Figures 3A, B**, **Tables S3**, **4**). Cyanidin is generally reported to be the most abundant anthocyanin of many fruits, among these, apple (Clayton-Cuch et al., 2023), pear (Ni et al., 2023) and chokeberry (Wen et al., 2020) mainly accumulated C3Ga (cyanidin-3-*O*-galactoside), while citrus (Huang et al., 2019), peach (Cheng et al., 2014), litchi (Li et al., 2016) and longan (Yi et al., 2021) predominantly accumulated C3G (cyanidin-3-*O*-glucosid), and jujube (Shi et al., 2020) primarily accumulated cyanidin-3-*O*-rutinoside (C3R). Here, we discovered that C3Ga is the most abundant anthocyanin in EH, followed by pelargonidin-3-*O*-galactoside (P3Ga), cyanidin-3-*O*-arabinoside (C3A) and C3G. On the contrary, all these anthocyanins were trace or undetectable in JFZ, and total anthocyanin content in EH was thousands of times higher than that in JFZ (**Figures 3C-E**).

As β-carotene and cyanidin-3-*O*-galactoside themselves show orange or red colors (Tanaka et al., 2008), the metabolic analysis here reveals that β-carotene and its derivatives contribute to orange flesh of cultivated loquat (**Figure 4**), while cyanidin-3-*O*-galactoside underlies red pigmentation of the wild species (**Figure 6**). The red appearance of wild loquat with high concentrations of anthocyanins is promising to meet current consumer expectations for novel color.

### Key carotenoid biosynthesis steps in loquat fruits

Carotenoids are derived from IPP and its allylic isomer DMAPP. The primary metabolic pathways of carotenoids have been widely studied in fruit crop and other horticulture plants (Nisar et al., 2015; Hermanns et al., 2020). Among dozens of carotenoid metabolic steps, PSY is regarded as a main rate-limiting enzyme, and variations in *Psy-A1*, *MePSY2* and *ClPSY1* resulted in greatly changes of total carotenoids content in wheat (He et al., 2007), cassava (Welsch et al., 2010) and watermelon (Liu et al., 2021). In addition, transcript levels of *PSY* homologues were also positively associated with fruit carotenoid content in citrus (Peng et al., 2013) and apple (Ampomah-Dwamena et al., 2015). In loquat, a segment deletion in C-terminal of *EjPSY2A* was discovered to cause lower carotenoid accumulation in fruits of white-fleshed loquat varieties (Fu et al., 2014). In this study, both JFZ and EH showed dominant genotype in *EjPSY2A* locus (**Figure 5A**), while the white *EjPSY2A* transcript level in JFZ was more than 11-fold higher than that in EH (**Figure 5C**). This suggests that low transcript level of key carotenoid biosynthesis gene acts as another candidate mechanism for weak carotenoid-pigmentation of wild loquat fruit.

Compared to EH, *ZDS* (*ζ-Carotene desaturase*) and *ZEP* (*zeaxanthin epoxidase*) expression levels in JFZ were more than 7-fold higher during fruits coloration (**Figures 4**, **5B, C**). As a key enzyme in the carotenoid biosynthesis pathway, ZDS can catalyze ζ-carotene to form lycopene. *EjZDS* was also up-regulated during fruit pigmentation of the orange-colored ‘Obusa’ loquat (Hadjipieri et al., 2017). Overexpression of apple *MdZDS* notably improved both carotenoid biosynthesis and saline–alkali stress tolerance in transgenic plants (Wang et al., 2023). In addition, ZEP paralog in yellow−fleshed sweet potato promoted carotenoid accumulation through the epoxidation of β-carotene and β-cryptoxanthin (Suematsu et al., 2020). While mutation in *CaZEP* contributes to orange coloration by improving carotenoid contents in pepper fruit (Lee et al., 2021).

On the other hand, NCED/CCD family proteins include NCEDs, CCD7, CCD8, CCD4, and CCD1 function in degradation of carotenoids into apocarotenoids. Carotenoid levels were negatively correlated with *NCED*/*CCD* expression, and natural variations in *PpCCD4*, *CrCCD4b* and *SiCCD1* strongly enhanced carotenoid content in peach (Falchi et al., 2013), citrus (Zheng et al., 2019) and millet (He et al., 2022). Corresponding to the very low carotenoid levels in white-fleshed fruit (**Figure 2**), transcript levels of NCED/CCDs in white-fleshed EH were 7.88 to 460.77 folds higher to that in orange-fleshed JFZ during fruit coloring (**Figure 5C**). Collectively, the strongly positive correlations of key biosynthesis gene expression patterns and negative correlations of metabolic gene expression patterns with carotenoid level changes in the loquat fruits suggest that *PSY*, *ZDS*, *ZEP* and *NCED*/*CCD* may be key biosynthesis/metabolic genes in loquat carotenoid accumulation.

### Key steps for anthocyanin biosynthesis in wild loquat fruits

Generally, anthocyanins biosynthesis can be divided into the early general phenylpropanoid pathway and the late flavonoid pathway (**Figure 6**). The key enzymes in the phenylpropanoid pathway, PAL, C4H and 4CL, convert phenylalanine to 4-coumaroyl-CoA (Tanaka et al., 2008). Then, 4-coumaroyl-CoA combines malonyl-CoA are catalyzed by a series of enzymes (CHS, CHI, F3H, F3’H, F3’5’H, DFR, ANS, UGT, etc.) to synthesize anthocyanin and other flavonoid compounds (Tanaka et al., 2008). UGT78 is the last and key enzyme for the biosynthesis of cyanidin-3-*O*-galactoside and pelargonidin-3-*O*-galactoside (**Figure 6**). VvGT6 (Ono et al., 2010), CsUGT78A15 (He et al., 2021), MrUGT78W1 (Ren et al., 2022) and MdUGT78T2 (Clayton-Cuch et al., 2023) all function in transferring galactosyl from UDP-Gal to flavonols to produce quercetin 3-*O*-galactoside and cyanidin-3-*O*-galactoside in grape, tea, waxberry and apple. EH accumulated a large amount of cyanidin-3-*O*-galactoside in its fruits (**Figure 3**). Correspondingly, notably high UGT78 transcription was detected in this species (**Figures 6**, **7**). ANS is a key enzyme at the end of the plant anthocyanin biosynthetic pathway that catalyzing the colorless leucoanthocyanidins into red-colored cyanidins. In this study, we identified two *ANS* homologues in loquat genome, and one of them (*Ej00061364*) highly expressed in red-colored EH, while it was undetectable in JFZ during fruit coloring (**Figures 6**, **7**). Mutations in the coding region of *RiANS* and *SmeFAS* resulted in loss of function of ANS protein and leads to loss of anthocyanin pigments in raspberry fruit (Rafique et al., 2016) and eggplant flower (Chen et al., 2018). On the other hand, overexpression of *ANS* dramatically elevated anthocyanin concentration in strawberry fruit (Giampieri et al., 2018) and silencing of *SlANS* expression significantly decreased anthocyanin accumulation in tomato (Sun et al., 2023).

Moreover, the expression levels of many enzyme-encoding genes upstream of *ANS* were also notably upregulated in EH as fruits initiated coloring (**Figures 6**, **7**). Among these, *CHS* and *CHI* were the mostly upregulated at both color breaker and ripen stages (7.5~27.0 and 8.6~105.7 folds compared to JFZ, see in **Figure 7**). CHS is the first committed step of flavonoid biosynthesis. Tobacco plants constitutively expressing *McCHS* (isolated from crabapple) displayed a higher anthocyanins accumulation and a deeper red petal color (Tai et al., 2014). Fruit collected from *CHS*-silenced apple line lacked flavonoids in the skin and flesh (Dare et al., 2013). *DcCHI1* (Zhu et al., 2021) or *CnCHI4* (Yu et al., 2022) overexpression significantly increased flavonoid production in tobacco. Furthermore, the red-pigmented EH also increased *F3H* and *F3’H* transcript levels to 4.4~9.9 or 4.4~22.6 folds higher than that in JFZ (**Figure 7**). Up-regulating the expression of *CitF3H* improves anthocyanin accumulation in blood orange (Ma et al., 2023), oppositely, mutation in *FvF3H* blocks anthocyanin biosynthesis and results in pink strawberry fruits (Xu et al., 2023). *DlF3′H* plays important role in selecting which anthocyanins component to be accumulated in red longan pericarp (Yi et al., 2021), loss of function of the *F3’H* (*tt7*) in *Arabidopsis* restricts catalyzation from dihydrokaempferol to dihydroquercetin, and leads to overaccumulation of kaempferol-3-rhamnoside in seed coat to compromise seed longevity (Niñoles et al., 2023). Totally, gene expression assays here demonstrate that *UGT78* (Ej00057656), *ANS* (Ej00061364), *F3H* (Ej00042569), *F3’H* (Ej00065084), *CHS* (Ej00054582) and *CHI* (Ej00071798) play crucial roles in anthocyanin biosynthesis flux determining in the red-colored wild loquat.

## Conclusion

In this study, we used fruits of an orange-colored loquat cultivar (JFZ) and a red-colored wild species (EH) to conduct carotenoid- and anthocyanin-targeted metabolomics analysis and transcriptome sequencing. The results showed that carotenoids including β-carotene (43.96%), violaxanthin palmitate (14.25%), rubixanthin laurate (5.26%), β-cryptoxanthin laurate (5.25%) and β-cryptoxanthin (5.08%) were the core metabolites leading to the orange colored fruits of JFZ. *PSY*, *ZDS* and *ZEP* were the key candidate genes responsible for carotenoid accumulation. Cyanidin-3-*O*-galactoside (96.75%), cyanidin-3-*O*-arabinoside, cyanidin-3-*O*-glucoside and pelargonidin-3-*O*-galactoside were the predominant anthocyanins contributed to the red pigmentation of the wild loquat fruits. Up-regulation of *ANS*, *UGT78*, *F3H*, *F3’H*, *CHS* and *CHI* expressions was tightly associated with anthocyanin content elevation in the red-colored EH fruits. In addition, these data implies that carotenoids might be positively selected during loquat domestication.

## Data availability statement

The original contributions presented in the study are included in the article/**Supplementary Material**, further inquiries can be directed to the corresponding authors.

## Author contributions

WS and JJ designed the research and obtained the funding. WS, ZF, SZ and YC performed fruit sample collection. WS, CZ, CD, YZ and XC extracted and assessed the anthocyanin and carotenoid metabolites. HL, ZT and WS performed the RT-qPCR. YK and WS performed genotyping of the loquat accessions used in this study. MH analyzed the RNA-seq data. WS and ZF analyzed the other data. WS, CZ, JJ, HL and CX prepared the manuscript. All authors contributed to the article and approved the submitted version.
