# Supplementary material for: Comprehensive metabolome and transcriptome analyses demonstrate divergent anthocyanin and carotenoid accumulation in fruits of wild and cultivated loquats
Source: Front Plant Sci. 2023 Oct 13;14:1285456. doi: 10.3389/fpls.2023.1285456 (PMC10611460; doi:10.3389/fpls.2023.1285456)
Supplement: Supplementary file 1 [file DataSheet_1.docx]

**Figure S1.** Transcriptome data used to identify DEGs contribute to carotenoid and anthocyanin accumulation of wild and cultivated loquat fruits.

**
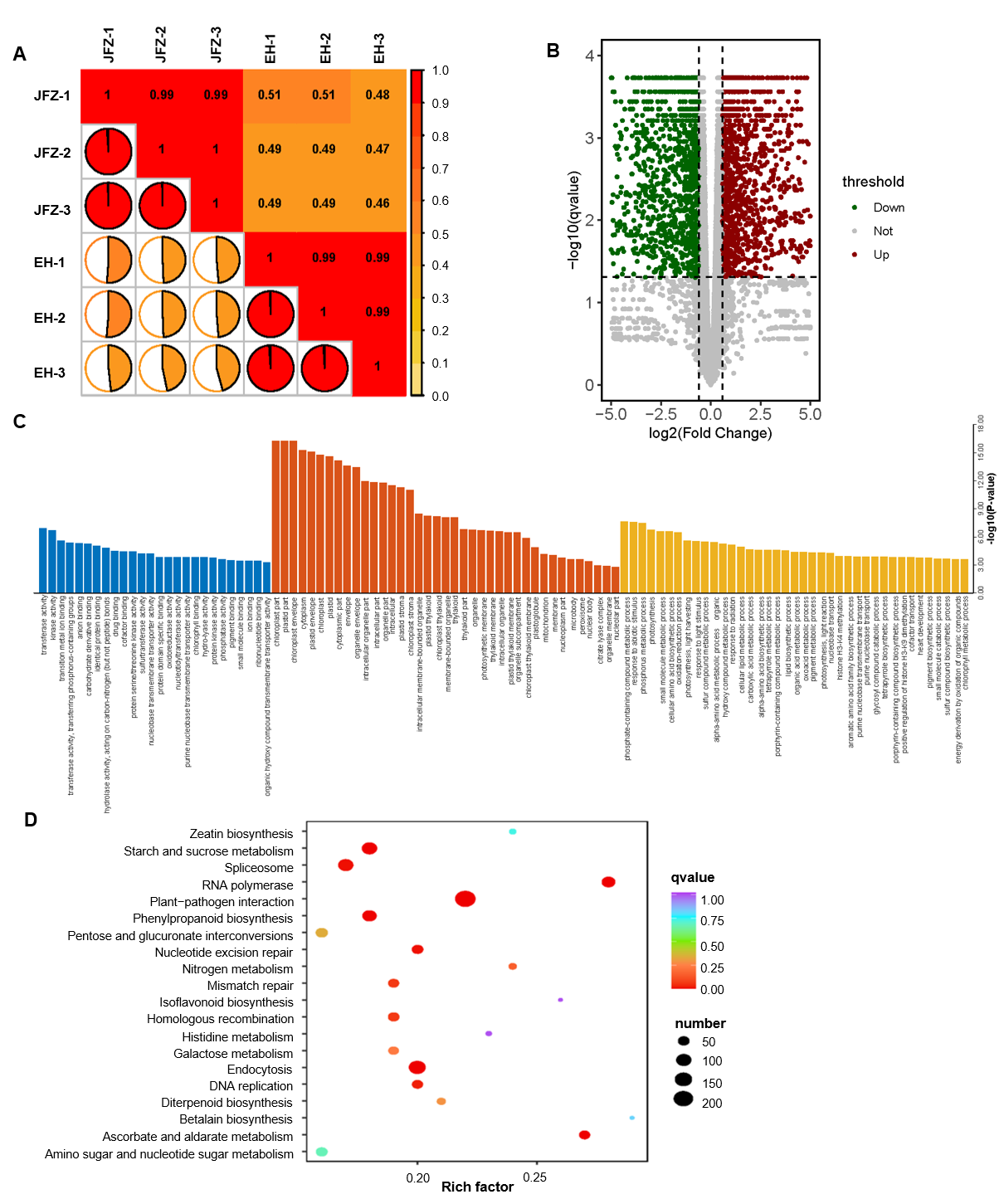
**

**Figure S2.** Heat map of carotenoid biosynthesis pathway genes in ripen fruits of wild and cultivated loquat.


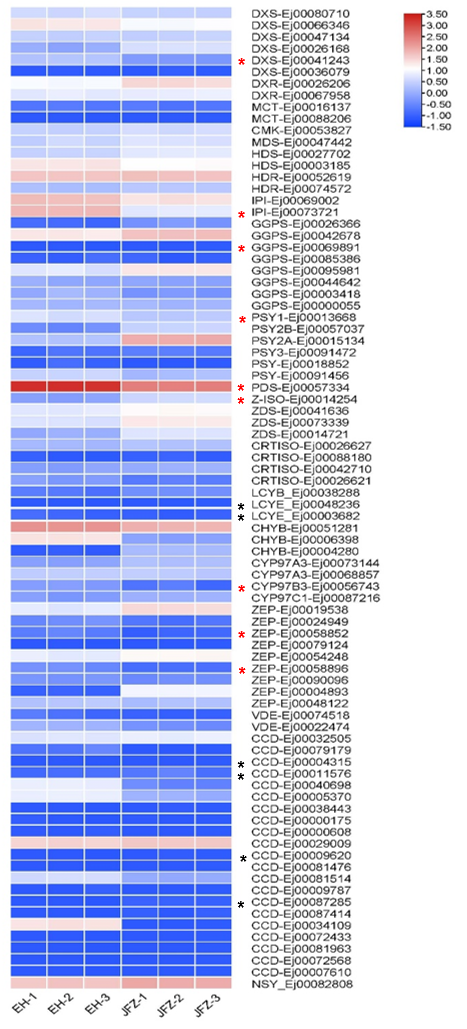


**Figure S3.** Heat map of anthocyanin biosynthesis pathway genes in ripen fruits of wild and cultivated loquat.


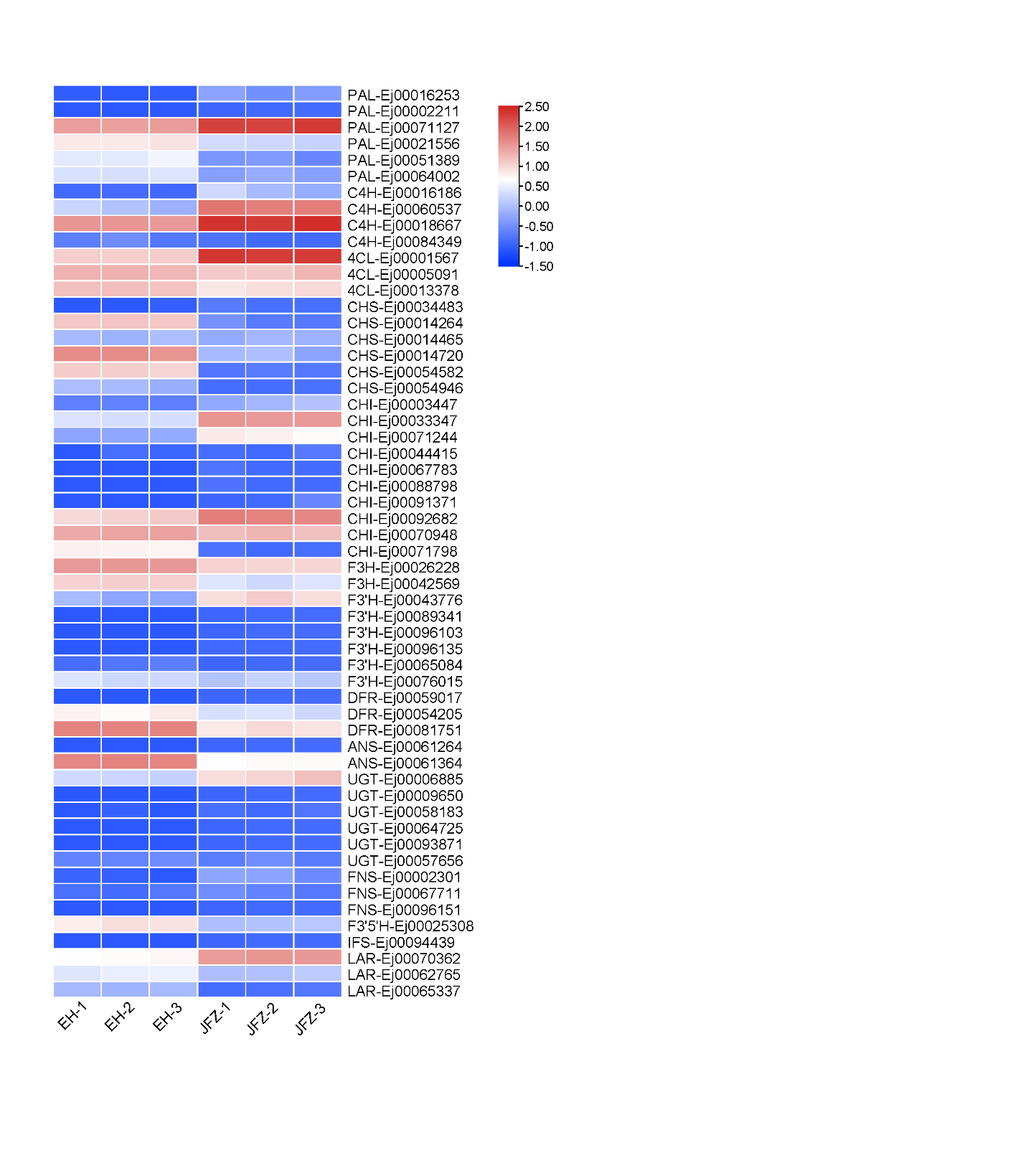


**Table S1** Linear equations of standard compounds for carotenoid quantification.

| **Index** | **Class** | **Equation** | **r** |
| --- | --- | --- | --- |
| Carotenoid_06 | carotenes | y = 0.31801 x + 0.06991 | 0.99427 |
| Carotenoid_04 | carotenes | y = 3.88371 x - 0.00451 | 0.99691 |
| Carotenoid_03 | carotenes | y = 2.63151 x - 0.00395 | 0.99632 |
| Carotenoid_57 | xanthophylls | y = 19.21851 x + 0.01079 | 0.99066 |
| Carotenoid_55 | xanthophylls | y = 3.08713 x + 0.00587 | 0.99127 |
| Carotenoid_59 | xanthophylls | y = 4.09580 x + 0.00722 | 0.99114 |
| Carotenoid_62 | xanthophylls | y = 12.62865 x - 3.96567e-4 | 0.99048 |
| Carotenoid_56 | xanthophylls | y = 1.55523 x + 0.02137 | 0.99831 |
| Carotenoid_66 | xanthophylls | y = 185.12097 x + 0.05837 | 0.99648 |
| Carotenoid_60 | xanthophylls | y = 2.86672 x + 0.01271 | 0.99189 |
| Carotenoid_67 | xanthophylls | y = 104.84245 x + 0.00241 | 0.99077 |
| Carotenoid_28 | xanthophylls | y = 9.58998 x + 0.00231 | 0.99263 |
| Carotenoid_09 | xanthophylls | y = 9.58998 x + 0.00231 | 0.99263 |
| Carotenoid_29 | xanthophylls | y = 9.58998 x + 0.00231 | 0.99263 |
| Carotenoid_30 | xanthophylls | y = 9.58998 x + 0.00231 | 0.99263 |
| Carotenoid_32 | xanthophylls | y = 9.58998 x + 0.00231 | 0.99263 |
| Carotenoid_23 | xanthophylls | y = 9.58998 x + 0.00231 | 0.99263 |
| Carotenoid_34 | xanthophylls | y = 9.58998 x + 0.00231 | 0.99263 |
| Carotenoid_33 | xanthophylls | y = 9.58998 x + 0.00231 | 0.99263 |
| Carotenoid_12 | xanthophylls | y = 9.58998 x + 0.00231 | 0.99263 |
| Carotenoid_51 | xanthophylls | y = 9.58998 x + 0.00231 | 0.99263 |
| Carotenoid_24 | xanthophylls | y = 9.58998 x + 0.00231 | 0.99263 |
| Carotenoid_39 | xanthophylls | y = 9.58998 x + 0.00231 | 0.99263 |
| Carotenoid_41 | xanthophylls | y = 9.58998 x + 0.00231 | 0.99263 |
| Carotenoid_54 | xanthophylls | y = 9.58998 x + 0.00231 | 0.99263 |
| Carotenoid_14 | xanthophylls | y = 9.58998 x + 0.00231 | 0.99263 |
| Carotenoid_52 | xanthophylls | y = 9.58998 x + 0.00231 | 0.99263 |
| Carotenoid_36 | xanthophylls | y = 9.58998 x + 0.00231 | 0.99263 |
| Carotenoid_15 | xanthophylls | y = 9.58998 x + 0.00231 | 0.99263 |
| Carotenoid_20 | xanthophylls | y = 9.58998 x + 0.00231 | 0.99263 |
| Carotenoid_25 | xanthophylls | y = 9.58998 x + 0.00231 | 0.99263 |
| Carotenoid_37 | xanthophylls | y = 9.58998 x + 0.00231 | 0.99263 |
| Carotenoid_53 | xanthophylls | y = 9.58998 x + 0.00231 | 0.99263 |
| Carotenoid_26 | xanthophylls | y = 9.58998 x + 0.00231 | 0.99263 |
| Carotenoid_17 | xanthophylls | y = 9.58998 x + 0.00231 | 0.99263 |
| Carotenoid_18 | xanthophylls | y = 9.58998 x + 0.00231 | 0.99263 |
| Carotenoid_19 | xanthophylls | y = 9.58998 x + 0.00231 | 0.99263 |
| Carotenoid_48 | xanthophylls | y = 17.44435 x + 0.05158 | 0.99340 |

**Table S2** Carotenoid compounds level (μg·g^-1^ DW) in peel of loquat fruits detected in this study.

| **Class** | **Index** | **Compounds** | **EH** | **SE(EH)** | **JFZ** | **SE(JFZ)** |
| --- | --- | --- | --- | --- | --- | --- |
| carotenes | Carotenoid_03 | γ-carotene | 0.07 | 0.00 | 0.81 | 0.04 |
| carotenes | Carotenoid_04 | β-carotene | 13.83 | 0.82 | 180.56 | 4.28 |
| carotenes | Carotenoid_06 | (E/Z)-phytoene | N/A | N/A | 7.18 | 0.52 |
| xanthophylls | Carotenoid_30 | violaxanthin palmitate | 1.91 | 0.14 | 58.53 | 0.98 |
| xanthophylls | Carotenoid_36 | violaxanthin-myristate-palmitate | 0.01 | 0.00 | 0.11 | 0.00 |
| xanthophylls | Carotenoid_34 | violaxanthin-myristate-laurate | N/A | N/A | 3.24 | 0.06 |
| xanthophylls | Carotenoid_33 | violaxanthin-myristate-caprate | 0.42 | 0.01 | 7.62 | 0.06 |
| xanthophylls | Carotenoid_29 | violaxanthin myristate | 0.80 | 0.02 | 3.05 | 0.01 |
| xanthophylls | Carotenoid_28 | violaxanthin laurate | 0.23 | 0.01 | N/A | N/A |
| xanthophylls | Carotenoid_37 | violaxanthin dipalmitate | N/A | N/A | 0.36 | 0.02 |
| xanthophylls | Carotenoid_48 | zeaxanthin dipalmitate | N/A | N/A | 1.53 | 0.01 |
| xanthophylls | Carotenoid_41 | zeaxanthin palmitate | 0.03 | 0.00 | 2.82 | 0.04 |
| xanthophylls | Carotenoid_60 | β-cryptoxanthin | 1.22 | 0.15 | 20.85 | 0.40 |
| xanthophylls | Carotenoid_51 | β-cryptoxanthin laurate | 1.04 | 0.15 | 21.57 | 0.68 |
| xanthophylls | Carotenoid_52 | β-cryptoxanthin myristate | 0.21 | 0.00 | 7.01 | 0.25 |
| xanthophylls | Carotenoid_54 | β-cryptoxanthin oleate | 0.03 | 0.00 | 8.06 | 0.30 |
| xanthophylls | Carotenoid_53 | β-cryptoxanthin palmitate | 0.29 | 0.08 | 14.19 | 0.21 |
| xanthophylls | Carotenoid_39 | violaxanthin dioleate | 0.03 | 0.01 | 0.25 | 0.02 |
| xanthophylls | Carotenoid_32 | violaxanthin dilaurate | 0.13 | 0.02 | 3.51 | 0.05 |
| xanthophylls | Carotenoid_14 | 5,6epoxy-luttein dilaurate | 0.02 | 0.00 | N/A | N/A |
| xanthophylls | Carotenoid_55 | antheraxanthin | 0.04 | 0.01 | N/A | N/A |
| xanthophylls | Carotenoid_62 | 8'-apo-beta-carotenal | 0.01 | 0.00 | 0.33 | 0.01 |
| xanthophylls | Carotenoid_59 | lutein | 9.02 | 0.23 | 4.49 | 0.06 |
| xanthophylls | Carotenoid_57 | violaxanthin | 0.46 | 0.05 | 0.50 | 0.01 |
| xanthophylls | Carotenoid_56 | zeaxanthin | 0.31 | 0.05 | N/A | N/A |
| xanthophylls | Carotenoid_66 | canthaxanthin | 0.00 | 0.00 | 0.01 | 0.00 |
| xanthophylls | Carotenoid_67 | echinenone | 0.01 | 0.00 | 0.46 | 0.00 |
| xanthophylls | Carotenoid_09 | lutein caprate | 0.05 | 0.00 | 0.15 | 0.01 |
| xanthophylls | Carotenoid_15 | lutein dilaurate | 0.25 | 0.02 | 8.89 | 0.23 |
| xanthophylls | Carotenoid_17 | lutein dimyristate | 0.11 | 0.01 | 3.42 | 0.05 |
| xanthophylls | Carotenoid_20 | lutein dioleate | 0.02 | 0.00 | 1.96 | 0.01 |
| xanthophylls | Carotenoid_18 | lutein dipalmitate | 0.08 | 0.00 | 5.15 | 0.08 |
| xanthophylls | Carotenoid_19 | lutein distearate | 0.03 | 0.00 | 4.35 | 0.11 |
| xanthophylls | Carotenoid_12 | lutein palmitate | 0.15 | 0.02 | 0.65 | 0.05 |
| xanthophylls | Carotenoid_23 | rubixanthin caprate | 0.15 | 0.01 | 1.53 | 0.03 |
| xanthophylls | Carotenoid_24 | rubixanthin laurate | 1.14 | 0.09 | 21.59 | 0.61 |
| xanthophylls | Carotenoid_25 | rubixanthin myristate | N/A | N/A | 0.08 | 0.00 |
| xanthophylls | Carotenoid_26 | rubixanthin palmitate | 0.38 | 0.02 | 15.89 | 0.71 |

**Table S3** Linear equations of standard compounds for anthocyanin quantification.

| **Index** | **Class** | | **Equation** | **r** | |
| --- | --- | --- | --- | --- | --- |
| Anthocyanidin_04 | | Cyanidin | y = 1.04213e5 x + 3143.47718 | | 0.99590 |
| Anthocyanidin_15 | | Cyanidin | y = 1.09504e5 x + 508.98433 | | 0.99770 |
| Anthocyanidin_11 | | Cyanidin | y = 1.21156e5 x + 12868.63817 | | 0.99822 |
| Anthocyanidin_14 | | Cyanidin | y = 1.53726e5 x + 3537.06445 | | 0.99206 |
| Anthocyanidin_08 | | Cyanidin | y = 2.16956e5 x + 2808.75385 | | 0.99302 |
| Anthocyanidin_16 | | Cyanidin | y = 1.35709e5 x + 4120.03033 | | 0.99695 |
| Anthocyanidin_05 | | Cyanidin | y = 1.39889e5 x + 2598.29613 | | 0.99080 |
| Anthocyanidin_33 | | Delphinidin | y = 3.15974e4 x - 502.35411 | | 0.99337 |
| Anthocyanidin_26 | | Delphinidin | y = 1.02135e5 x - 5198.25109 | | 0.99528 |
| Anthocyanidin_31 | | Delphinidin | y = 4.16141e4 x - 10497.29832 | | 0.99942 |
| Anthocyanidin_27 | | Delphinidin | y = 1.23549e5 x - 2817.84864 | | 0.99094 |
| Anthocyanidin_38 | | flavonoid | y = 2373.81586 x + 992.17831 | | 0.99552 |
| Anthocyanidin_34 | | flavonoid | y = 2426.55827 x - 2048.78298 | | 0.99728 |
| Anthocyanidin_39 | | flavonoid | y = 3.95185e4 x + 2719.00149 | | 0.99532 |
| Anthocyanidin_41 | | flavonoid | y = 5137.00819 x + 883.62145 | | 0.99496 |
| Anthocyanidin_42 | | flavonoid | y = 2178.31911 x + 1537.15370 | | 0.99757 |
| Anthocyanidin_40 | | flavonoid | y = 4599.88770 x - 466.78881 | | 0.99702 |
| Anthocyanidin_67 | | Pelargonidin | y = 4.16141e4 x - 10497.29832 | | 0.99942 |
| Anthocyanidin_68 | | Pelargonidin | y = 1.78373e5 x + 741.76097 | | 0.99421 |
| Anthocyanidin_66 | | Pelargonidin | y = 4.16141e4 x - 10497.29832 | | 0.99942 |
| Anthocyanidin_86 | | Peonidin | y = 3.59047e5 x + 28340.95606 | | 0.99693 |
| Anthocyanidin_89 | | Peonidin | y = 4.16141e4 x - 10497.29832 | | 0.99942 |
| Anthocyanidin_75 | | Peonidin | y = 4.16141e4 x - 10497.29832 | | 0.99942 |
| Anthocyanidin_82 | | Peonidin | y = 2.61389e5 x + 1262.65215 | | 0.99168 |
| Anthocyanidin_94 | | Petunidin | y = 4.16141e4 x - 10497.29832 | | 0.99942 |
| Anthocyanidin_97 | | Petunidin | y = 4.16141e4 x - 10497.29832 | | 0.99942 |
| Anthocyanidin_98 | | Petunidin | y = 1.63873e5 x + 5311.65646 | | 0.99835 |
| Anthocyanidin_93 | | Petunidin | y = 4.16141e4 x - 10497.29832 | | 0.99942 |
| Anthocyanidin_100 | | Petunidin | y = 4.16141e4 x - 10497.29832 | | 0.99942 |
| Anthocyanidin_107 | | Procyanidin | y = 3468.14194 x + 424.78772 | | 0.99972 |
| Anthocyanidin_105 | | Procyanidin | y = 5765.71481 x + 168.39633 | | 0.99981 |
| Anthocyanidin_106 | | Procyanidin | y = 3321.67545 x - 492.14029 | | 0.99944 |
| Anthocyanidin_108 | | Procyanidin | y = 457.95677 x + 437.68616 | | 0.99191 |
| Anthocyanidin_104 | | Procyanidin | y = 8461.51423 x + 1052.06223 | | 0.99881 |

**Table S4** Anthocyanidin compounds content (μg·g^-1^ DW) in peel of loquat fruits detected in this study.

| **Class** | **Index** | **Compounds** | **EH** | **SE(EH)** | **JFZ** | **SE(JFZ)** |
| --- | --- | --- | --- | --- | --- | --- |
| Cyanidin | Anthocyanidin_11 | Cyanidin-3-O-glucoside | 2.4083 | 0.1262 | 0.0065 | 0.0003 |
|  | Anthocyanidin_15 | Cyanidin-3-O-sophoroside | 0.0876 | 0.0043 | 0.0022 | 0.0003 |
|  | Anthocyanidin_04 | Cyanidin-3,5-O-diglucoside | 0.0174 | 0.0005 | N/A | N/A |
|  | Anthocyanidin_05 | Cyanidin-3-O-(6-O-malonyl-beta-D-glucoside) | 0.0042 | 0.0002 | N/A | N/A |
|  | Anthocyanidin_16 | Cyanidin-3-O-xyloside | 0.1032 | 0.0036 | N/A | N/A |
|  | Anthocyanidin_08 | Cyanidin-3-O-arabinoside | 4.5667 | 0.0883 | N/A | N/A |
|  | Anthocyanidin_10 | Cyanidin-3-O-galactoside | 649.8643 | 17.7326 | N/A | N/A |
|  | Anthocyanidin_14 | Cyanidin-3-O-sambubioside | 0.0440 | 0.0013 | N/A | N/A |
| Flavonoid | Anthocyanidin_41 | Quercetin-3-O-glucoside | 10.7816 | 0.4244 | 40.3468 | 2.6846 |
|  | Anthocyanidin_34 | Afzelin | 58.0921 | 6.6634 | 73.8397 | 1.5201 |
|  | Anthocyanidin_38 | Kaempferol-3-O-rutinoside | 11.9027 | 1.8369 | 0.6966 | 0.0312 |
|  | Anthocyanidin_42 | Rutin | 24.9662 | 2.3007 | 8.6312 | 0.8095 |
|  | Anthocyanidin_39 | Naringenin | 0.0294 | 0.0020 | N/A | N/A |
|  | Anthocyanidin_40 | Naringenin-7-O-glucoside | N/A | N/A | 0.1288 | 0.0028 |
| Procyanidin | Anthocyanidin_104 | Procyanidin A2 | 0.9500 | 0.0700 | 0.0577 | 0.0038 |
|  | Anthocyanidin_105 | Procyanidin B1 | 9.5784 | 0.2205 | 5.3605 | 1.0171 |
|  | Anthocyanidin_106 | Procyanidin B2 | 85.4653 | 2.4166 | 75.0300 | 15.0375 |
|  | Anthocyanidin_107 | Procyanidin B3 | 3.5426 | 0.1664 | 0.3916 | 0.0453 |
|  | Anthocyanidin_108 | Procyanidin C1 | 68.9290 | 3.9503 | 14.5499 | 0.6366 |
| Delphinidin | Anthocyanidin_31 | Delphinidin-3-O-sophoroside | 0.0136 | 0.0005 | 0.0196 | 0.0023 |
|  | Anthocyanidin_33 | Delphinidin-3-O-sambubioside-5-O-glucoside | 0.0427 | 0.0027 | 0.0074 | 0.0007 |
|  | Anthocyanidin_27 | Delphinidin-3-O-glucoside | N/A | N/A | 0.0130 | 0.0004 |
|  | Anthocyanidin_26 | Delphinidin-3-O-galactoside | N/A | N/A | 0.0203 | 0.0027 |
| Peonidin | Anthocyanidin_82 | Peonidin-3-O-arabinoside | 0.0036 | 0.0002 | N/A | N/A |
|  | Anthocyanidin_75 | Peonidin | N/A | N/A | 0.0649 | 0.0020 |
|  | Anthocyanidin_86 | Peonidin-3-O-glucoside | 0.0032 | 0.0002 | N/A | N/A |
|  | Anthocyanidin_89 | Peonidin-3-O-sambubioside | N/A | N/A | 0.0116 | 0.0001 |
| Petunidin | Anthocyanidin_94 | Petunidin-3-O-(6-O-p-coumaroyl)-glucoside | 0.0268 | 0.0022 | 0.0096 | 0.0003 |
|  | Anthocyanidin_97 | Petunidin-3-O-galactoside | 0.0255 | 0.0011 | 0.0194 | 0.0019 |
|  | Anthocyanidin_98 | Petunidin-3-O-glucoside | 0.0020 | 0.0001 | N/A | N/A |
|  | Anthocyanidin_93 | Petunidin-3-O-(6-O-malonyl-beta-D-glucoside) | 0.0107 | 0.0003 | N/A | N/A |
| Pelargonidin | Anthocyanidin_68 | Pelargonidin-3-O-glucoside | 0.0688 | 0.0034 | N/A | N/A |
|  | Anthocyanidin_66 | Pelargonidin-3-O-arabinoside | 0.1419 | 0.0023 | N/A | N/A |
|  | Anthocyanidin_67 | Pelargonidin-3-O-galactoside | 14.3691 | 0.5931 | N/A | N/A |
| Total | | | 946.0409 | 25.4374 | 219.2075 | 21.1918 |

**Table S5** Basic sequencing data of ripen ‘Jiefangzhong’ and *Eriobotrya henryi* fruits.

| **Sample** | **Raw Reads** | **Clean Reads** | **Clean Base(G)** | **Error Rate (%)** | **Q20 (%)** | **Q30 (%)** | **GC Content (%)** |
| --- | --- | --- | --- | --- | --- | --- | --- |
| JFZ-1 | 52136296 | 49822838 | 7.47 | 0.03 | 97.89 | 93.8 | 47.23 |
| JFZ-2 | 52650460 | 51147438 | 7.67 | 0.03 | 97.98 | 93.99 | 47.08 |
| JFZ-3 | 46644410 | 45353540 | 6.8 | 0.03 | 97.85 | 93.64 | 47.14 |
| EH-1 | 47068116 | 44690950 | 6.7 | 0.03 | 97.77 | 93.65 | 47.11 |
| EH -2 | 49733916 | 47391062 | 7.11 | 0.03 | 97.68 | 93.42 | 47.26 |
| EH -3 | 46458180 | 44635768 | 6.7 | 0.03 | 97.89 | 93.91 | 46.87 |

**Table S6** Mapping rates of transcriptome sequencing data to reference loquat genome.

| **Sample** | **Total Reads** | **Reads mapped** | **Unique mapped** | **Multi mapped** | **Read1 mapped** | **Read2 mapped** | **'+' mapped** | **'-' mapped** |
| --- | --- | --- | --- | --- | --- | --- | --- | --- |
| JFZ-1 | 49822838 | 47580262(95.50%) | 45706830(91.74%) | 2945481(3.76%) | 22874542(45.91%) | 22832288(45.83%) | 22848336(45.86%) | 22858494(45.88%) |
| JFZ-2 | 51147438 | 48591522(95.00%) | 46624873(91.16%) | 3088504(3.85%) | 23317444(45.59%) | 23307429(45.57%) | 23312722(45.58%) | 23312151(45.58%) |
| JFZ-3 | 45353540 | 43000936(94.81%) | 41283918(91.03%) | 2717216(3.79%) | 20668461(45.57%) | 20615457(45.46%) | 20639019(45.51%) | 20644899(45.52%) |
| EH-1 | 44690950 | 35551010(79.55%) | 34229055(76.59%) | 2018847(2.96%) | 17207277(38.50%) | 17021778(38.09%) | 17128927(38.33%) | 17100128(38.26%) |
| EH -2 | 47391062 | 37965762(80.11%) | 36532293(77.09%) | 2180228(3.02%) | 18335151(38.69%) | 18197142(38.40%) | 18336386(38.69%) | 18195907(38.40%) |
| EH -3 | 44635768 | 35843995(80.30%) | 34473888(77.23%) | 2124479(3.07%) | 17290460(38.74%) | 17183428(38.50%) | 17293791(38.74%) | 17180097(38.49%) |

**Table S7** Carotenoid and anthocyanin biosynthesis structure genes identified in loquat genome.

| **Carotenoid biosynthesis structure genes** | | | | | | **Anthocyanin biosynthesis structure genes** | | |
| --- | --- | --- | --- | --- | --- | --- | --- | --- |
| **Gene symbol** | Loquat Gene ID | Arabidopsis homologue | **Gene symbol** | Loquat Gene ID | Arabidopsis homologue | **Gene symbol** | Loquat Gene ID | Arabidopsis homologue |
| DXS | Ej00080710 | AT4G15560 | VDE | Ej00074518 | AT1G08550 | PAL | Ej00016253 | AT2G37040 |
| DXS | Ej00066346 | AT4G15560 | VDE | Ej00022474 | AT1G08550 | PAL | Ej00002211 | AT2G37040 |
| DXS | Ej00047134 | AT4G15560 | CCD | Ej00032505 | AT3G63520 | PAL | Ej00071127 | AT2G37040 |
| DXS | Ej00026168 | AT4G15560 | CCD | Ej00079179 | AT3G63520 | PAL | Ej00021556 | AT2G37040 |
| DXS | Ej00041243 | AT4G15560 | CCD | Ej00004315 | AT3G63520 | PAL | Ej00051389 | AT2G37040 |
| DXS | Ej00036079 | AT4G15560 | CCD | Ej00011576 | AT3G63520 | PAL | Ej00064002 | AT2G37040 |
| DXR | Ej00026206 | AT5G62790 | CCD | Ej00040698 | AT3G63520 | C4H | Ej00016186 | AT2G30490 |
| DXR | Ej00067958 | AT5G62790 | CCD | Ej00005370 | AT3G63520 | C4H | Ej00060537 | AT2G30490 |
| HDS | Ej00027702 | AT5G60600 | CCD | Ej00038443 | AT3G63520 | C4H | Ej00018667 | AT2G30490 |
| HDS | Ej00003185 | AT5G60600 | CCD | Ej00000175 | AT3G63520 | C4H | Ej00084349 | AT2G30490 |
| HDR | Ej00052619 | AT4G34350 | CCD | Ej00000608 | AT3G63520 | 4CL | Ej00001567 | AT1G51680 |
| HDR | Ej00074572 | AT4G34350 | CCD | Ej00029009 | AT3G63520 | 4CL | Ej00005091 | AT1G51680 |
| IPI | Ej00069002 | AT5G16440 | CCD | Ej00009620 | AT3G63520 | 4CL | Ej00013378 | AT1G51680 |
| IPI | Ej00073721 | AT5G16440 | CCD | Ej00081476 | AT3G63520 | CHS | Ej00034483 | AT5G13930 |
| GGPS | Ej00026366 | AT1G49530 | CCD | Ej00081514 | AT3G63520 | CHS | Ej00014264 | AT5G13930 |
| GGPS | Ej00042678 | AT1G49530 | CCD | Ej00009787 | AT3G63520 | CHS | Ej00014465 | AT5G13930 |
| GGPS | Ej00069891 | AT1G49530 | CCD | Ej00087285 | AT3G63520 | CHS | Ej00014720 | AT5G13930 |
| GGPS | Ej00085386 | AT1G49530 | CCD | Ej00087414 | AT3G63520 | CHS | Ej00054582 | AT5G13930 |
| GGPS | Ej00095981 | AT1G49530 | CCD | Ej00034109 | AT3G63520 | CHS | Ej00054946 | AT5G13930 |
| GGPS | Ej00044642 | AT1G49530 | CCD | Ej00072433 | AT3G63520 | CHI | Ej00003447 | AT3G55120 |
| GGPS | Ej00003418 | AT1G49530 | CCD | Ej00081963 | AT3G63520 | CHI | Ej00033347 | AT3G55120 |
| GGPS | Ej00000055 | AT1G49530 | CCD | Ej00072568 | AT3G63520 | CHI | Ej00071244 | AT3G55120 |
| PSY | Ej00013668 | AT5G17230 | CCD | Ej00007610 | AT3G63520 | CHI | Ej00044415 | AT3G55120 |
| PSY | Ej00057037 | AT5G17230 | NSY | Ej00082808 | AT1G67080 | CHI | Ej00067783 | AT3G55120 |
| PSY | Ej00015134 | AT5G17230 |  |  |  | CHI | Ej00088798 | AT3G55120 |
| PSY | Ej00091472 | AT5G17230 |  |  |  | CHI | Ej00091371 | AT3G55120 |
| PSY | Ej00018852 | AT5G17230 |  |  |  | CHI | Ej00092682 | AT3G55120 |
| PSY | Ej00091456 | AT5G17230 |  |  |  | CHI | Ej00070948 | AT3G55120 |
| PDS | Ej00057334 | AT4G14210 |  |  |  | CHI | Ej00071798 | AT3G55120 |
| Z-ISO | Ej00014254 | AT1G10830 |  |  |  | F3H | Ej00026228 | AT3G51240 |
| ZDS | Ej00041636 | AT3G04870 |  |  |  | F3H | Ej00042569 | AT3G51240 |
| ZDS | Ej00073339 | AT3G04870 |  |  |  | F3'H | Ej00043776 | AT3G51240 |
| ZDS | Ej00014721 | AT3G04870 |  |  |  | F3'H | Ej00089341 | AT3G51240 |
| CRTISO | Ej00026627 | AT1G06820 |  |  |  | F3'H | Ej00096103 | AT3G51240 |
| CRTISO | Ej00088180 | AT1G06820 |  |  |  | F3'H | Ej00096135 | AT3G51240 |
| CRTISO | Ej00042710 | AT1G06820 |  |  |  | F3'H | Ej00065084 | AT3G51240 |
| CRTISO | Ej00026621 | AT1G06820 |  |  |  | F3'H | Ej00076015 | AT3G51240 |
| LCYB | Ej00038288 | AT2G32640 |  |  |  | DFR | Ej00059017 | AT5G42800 |
| LCYE | Ej00048236 | AT3G10230 |  |  |  | DFR | Ej00054205 | AT5G42800 |
| LCYE | Ej00003682 | AT3G10230 |  |  |  | DFR | Ej00081751 | AT5G42800 |
| BCH | Ej00051281 | AT5G52570 |  |  |  | ANS | Ej00061264 | AT4G22880 |
| BCH | Ej00006398 | AT5G52570 |  |  |  | ANS | Ej00061364 | AT4G22880 |
| BCH | Ej00004280 | AT5G52570 |  |  |  | UGT78 | Ej00006885 | AT1G30530 |
| CYP97A | Ej00073144 | AT1G31800 |  |  |  | UGT78 | Ej00009650 | AT1G30530 |
| CYP97A | Ej00068857 | AT1G31800 |  |  |  | UGT78 | Ej00058183 | AT1G30530 |
| CYP97B | Ej00056743 | AT4G15110 |  |  |  | UGT78 | Ej00064725 | AT1G30530 |
| CYP97C | Ej00087216 | AT3G53130 |  |  |  | UGT78 | Ej00093871 | AT1G30530 |
| ZEP | Ej00019538 | AT5G67030 |  |  |  | UGT78 | Ej00057656 | AT1G30530 |
| ZEP | Ej00024949 | AT5G67030 |  |  |  | FNS | Ej00002301 | AT5G18810 |
| ZEP | Ej00058852 | AT5G67030 |  |  |  | FNS | Ej00067711 | AT5G18810 |
| ZEP | Ej00079124 | AT5G67030 |  |  |  | FNS | Ej00096151 | AT5G18810 |
| ZEP | Ej00054248 | AT5G67030 |  |  |  | F3'5'H | Ej00025308 | AT5G07990 |
| ZEP | Ej00058896 | AT5G67030 |  |  |  | LAR | Ej00070362 | AT4G22880 |
| ZEP | Ej00090096 | AT5G67030 |  |  |  | LAR | Ej00062765 | AT4G22880 |
| ZEP | Ej00004893 | AT5G67030 |  |  |  | LAR | Ej00065337 | AT4G22880 |
| ZEP | Ej00048122 | AT5G67030 |  |  |  |  |  |  |

**Table S8** Primers used for Q-RT-PCR in this study.

| **Purpose** | **Primer** | **Forward primer (5′→3′)** | **Reverse primer (5′→3′)** |
| --- | --- | --- | --- |
| Carotenoid metabolism | Ej00026206 | AACTGGGATGGCCTGATA | GGTCCATGGATGGGTATTTC |
|  | Ej00042678 | CTCGACGTGACCAAATCTTC | TGGACTTCTCGATCCCTAAC |
|  | Ej00095981 | CCCTCGAACCACACAATTTA | CAATGTGCTGAAACCCTAATG |
|  | Ej00015134 | GGATATGCCCAGAATCAAG | CTTCCCGCGAAAGATGTCATC |
|  | Ej00041636 | CCATCATCCCACGGTTTAG | CTCAATCCCACCAACTCTTC |
|  | Ej00073339 | CACCACTACGACTGACTTTAC | CCACTGCATTTCTTGCTTTATC |
|  | Ej00019538 | GTTGGAGCCGATGGTATATG | ACTCGGTACCCTACAGAATTA |
|  | Ej00054248 | GAGTGGATGATGTGGTGATAG | GGTTCTGAGGCCGTCTGATTTC |
|  | Ej00004893 | CGTTCATGTTATCGGAGGTTAG | CGAACCAGTAGACCAAGTTATC |
|  | Ej00040698 | ACCCGGTTCGCTTATTTG | GTACTTCTCCTCGCCATAGA |
|  | Ej00005370 | GGTGAGGTGAAGAAGCATATC | TAACCATCGTCCTCGTTCT |
|  | Ej00081514 | GGAGACCTTGTTGAGGTAATC | GTCTACCCAAGTCTGGTTTG |
|  | Ej00034109 | CATGCTTCACTCCGTTAGG | GAAGGCGTTGGGAAGTATAG |
| Anthocyanin  biosynthesis | Ej00061364 | GTGTCAGTGCCTGTTATCTC | GGATCAAAGACGACGGTATG |
|  | Ej00081751 | GACTGTTGGACATCCTGAAG | CTCCTCCACATTCACAGTTC |
|  | Ej00065084 | GCTGTCTCTCAAGGATGATGC | GAATGAGTTCTGCTATGGCCC |
|  | Ej00076015 | GACATGGTTGATGTGCTACT | CTTGAATACCTCTGGCTTCC |
|  | Ej00026228 | GGGAGGTGACGAAGAAGTA | AGGGCACTTCGGATAGAA |
|  | Ej00042569 | GTGGACATGGACCAGAAAG | GTCTTGAAGCAGAAGGGTAAT |
|  | Ej00014264 | GTAGGGCTTACATTTCAC | GTTGCTTCTAGTTTCTCC |
|  | Ej00014720 | GAGAAGCTAGAAGCTACA | CCAAATCCAAAGAGTACG |
|  | Ej00054582 | CTTACGTTTCACCTTCTG | CTTGTCGCTTCTAGTTTC |
|  | Ej00070948 | GGCACTGAAGTTGTTATG | CTTCAGGGTCCAAGTAAA |
|  | Ej00071798 | TCCTCCAATACTCTGTTC | GTCTTACCCTTCCACTTA |
|  | Ej00006885 | CACTCTCTTCTCGTTCTT | CATCGTACACCCTTATGT |
|  | Ej00057656 | GTTGGAAGCCTGTTATAC | CTGCCAGTATCATCTTTG |
| House keeping | EjACT2 | CTTTCCCTCTATGCCAGTG | CAAGGTCAAGCCTCAAGAT |
